# TRIM11 activates the proteasome and promotes overall protein degradation by regulating USP14

**DOI:** 10.1038/s41467-018-03499-z

**Published:** 2018-03-26

**Authors:** Liang Chen, Guixin Zhu, Eleanor M. Johns, Xiaolu Yang

**Affiliations:** Department of Cancer Biology and Abramson Family Cancer Research Institute, Perelman School of Medicine, University of Pennsylvania, Philadelphia, PA 19104 USA

## Abstract

The proteasome is a complex protease critical for protein quality control and cell regulation, and its dysfunction is associated with cancer and other diseases. However, the mechanisms that control proteasome activity  in normal and malignant cells remain unclear. Here we report that TRIM11 enhances degradation of aberrant and normal regulatory proteins, and augments overall rate of proteolysis. Mechanistically, TRIM11 binds to both the proteasome and USP14, a deubiquitinase that prematurely removes ubiquitins from proteasome-bound substrates and also noncatalytically inhibits the proteasome, and precludes their association, thereby increasing proteasome activity. TRIM11 promotes cell survival and is upregulated upon heat shock. Moreover, TRIM11 is required for tumor growth, and increased expression of TRIM11 correlates with poor clinical survival. These findings identify TRIM11 as an important activator of the proteasome, define a pathway that adjusts proteasome activity, and reveal a mechanism by which tumor cells acquire higher degradative power to support oncogenic growth.

## Abstract (web summary)

The proteasome-bound ubiquitinase USP14 plays an important role in determining proteasome activity and substrate specificity. Here the authors show that TRIM11, a member of the mammalian tripartite motif family, regulates USP14 and is an important activator of the proteasome.

## Introduction

Intracellular protein degradation is fundamental for protein quality control and cell regulation. Terminally misfolded and damaged proteins, which are prevalent due to the inherent difficulty in protein folding and the crowded and reactive cellular environment, need to be recycled to avoid toxicity^1^. Normal, correctly folded proteins often require controlled degradation to achieve levels appropriate for the regulation of cell cycle, apoptosis, inflammation, and various other cellular processes. Central to the proteolysis of both aberrant and normal regulatory proteins is the ubiquitin-proteasome pathway (UPS). Protein ubiquitination involves three sequential enzymatic steps performed by ubiquitin activating enzymes (E1s), ubiquitin-conjugating enzymes (E2s), and ubiquitin ligases (E3s), respectively^2^, with its specificity and efficiency largely determined by the numerous ubiquitin ligases, each recognizing a specific set of substrates.

The degradation of ubiquitinated proteins occurs in the proteasome, the most complex protease known^3–5^. The holoenzyme (the 26S proteasome) has a size over 2.5 MDa consisting of at least 47 subunits. These subunits are organized into the 28-subunit 20S catalytic core particle (CP) and the 19-subunit 19S regulatory particle (RP, also known as PA700). The CP is a hollow cylindrical structure with four stacked heptameric rings formed by either α- (outer ring) or β- (inner ring) type subunits, which sequester proteolytic active sites of distinct specificities within the internal chamber. The CP is capped at one or both ends by the RP, which prepares ubiquitinated proteins for degradation. The CP has ubiquitin receptors that dock ubiquitinated substrates, a degradation-coupled deubiquitinase that cleaves the ubiquitin chains off of substrates once they are committed to degradation, and several ATPases that unfold and transport the substrates to the internal chamber of the CP for hydrolysis.

The fate of the ubiquitinated proteins, however, can be reversed through the action of other proteasome-associated deubiquitinases, notably ubiquitin-specific protease 14 (USP14, known in yeast as Ubp6)^4,5^. USP14 binds to the proteasome via the 19S subunit PSMD2/Rpn1 (ref. ^6^) and usually at substoichiometric amounts^7^, and its recruitment is enhanced by the presence of ubiquitinated proteins on the proteasome^8^. Upon binding to the proteasome, USP14 becomes activated and removes ubiquitin chains en bloc from a potential substrate^9^, reducing its residence time on the proteasome and allowing it to escape degradation^10,11^. USP14 additionally employs a non-catalytic mechanism likely by inducing conformational changes in proteasome subunits^12–14^, leading to the inhibition of multiple proteasomal activities^15^. Inhibition of USP14 by a small molecule enhances proteasome activity and decreases misfolded proteins in mammalian cells following proteotoxic stress^11^. However, despite increasing recognition of the importance of USP14 in regulating the proteasome, the cellular mechanism that controls USP14 remains largely undefined. Especially, it is unclear how USP14 may be counteracted to bolster proteasome function and enable its adaptation to increased demand for proteolysis.

Tripartite motif (TRIMs) proteins constitute a distinct family in metazoans characterized by an N-terminal TRIM/RBCC motif comprising a RING domain, one or two B-Boxes, and a predicted coiled-coil region, which is followed by a more diverse C-terminal region^16,17^. Many TRIMs are ubiquitin E3 ligases that mediate the degradation of normal regulatory proteins^17,18^. Emerging evidence suggests that TRIMs can also promote the degradation of aberrant proteins, a property attributable to SUMO (small ubiquitin-like (UBL) modifier) E3 ligase activity^19^. For example, PML (also known as TRIM19) recognizes a variety of misfolded proteins in the nucleus, and marks them with poly-SUMO2/3 chains^20^. This permits misfolded proteins to be ubiquitinated by SUMO-targeted ubiquitin ligases and subsequently degraded in the proteasome^20^. TRIM11 appears to share this ability, and it functions in the cytoplasm as well as the nucleus^21^. By removing misfolded proteins, TRIMs may protect against neurodegeneration^20^. In contrast, upregulation of TRIM11 and other TRIMs may contribute to an enhanced cellular capacity to remove misfolded proteins in tumor cells, which enables robust anti-oxidant defense and oncogenic growth^21^. Still, the mechanisms by which TRIMs regulate protein degradation and their functions in human diseases are not well characterized.

Here, we investigate the role of TRIM11 in global protein degradation, its mechanism of action, and its involvement in tumorigenesis. We find that TRIM11 enhances the degradation of both misfolded and normal regulatory proteins, and augments overall rate of proteolysis. TRIM11 directly activates proteasome. It binds to both the proteasome and USP14, preventing their association and counteracting the inhibitory effect of USP14 on the proteasome. The expression of TRIM11 is upregulated by heat shock to permit cellular adaptation to proteotoxic stress. TRIM11 is upregulated in colon cancer cells, and it may be co-opted by tumor cells to promote oncogenic growth. These results define a previously unrecognized role of TRIM proteins in promoting proteolysis and maintaining protein homeostasis, and reveal a mechanism underlying increased proteasome activity in tumor cells.

## Results

### TRIM11 promotes the degradation of misfolded proteins

**Fig. 1:**
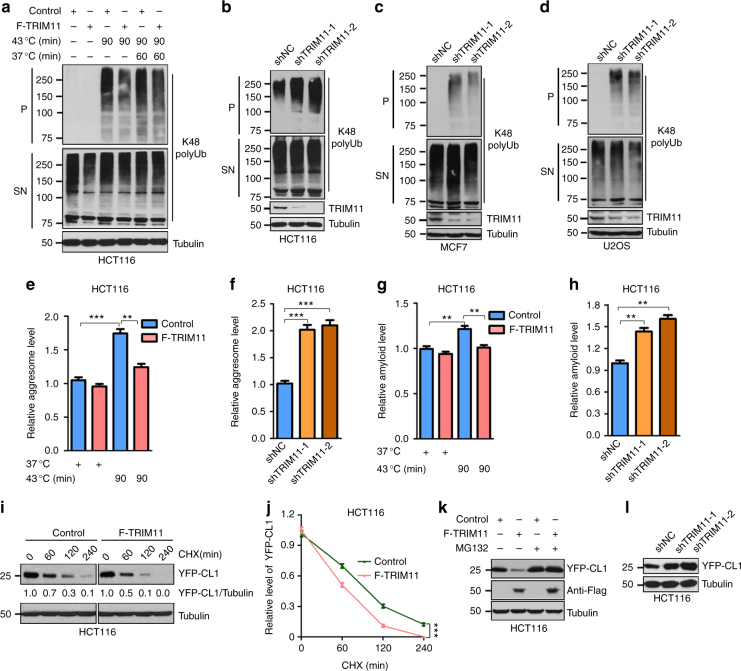
TRIM11 promotes degradation of misfolded proteins. **a** HCT116 cells infected with control or Flag-TRIM11 (F-TRIM11) lentiviruses were grown at 37 °C (−), heat shocked at 43 °C for 90 min, or heat shocked and then recovered at 37 °C for 60 min. Cells were lysed in buffer containing non-ionic detergent. Cell lysates were separated into supernatant (SN) and pellet (P) fractions by centrifugation and analyzed by western blot. Molecular weight markers (in kDa) are indicated on the left. **b**–**d** Levels of K48 polyUb conjugates in HCT116 (**b**), MCF7 (**c**), and U2OS (**d**) cells expressing a control (shNC) or TRIM11 shRNA. **e**,** g** Relative levels of aggresomes (**e**) and amyloid fibril (**g**) in HCT116 cells infected with control or Flag-TRIM11 lentiviruses and grown under unstressed or heat shock condition. **f**,** h** Relative levels of aggresomes (**f**) and amyloid fibril (**h**) in HCT116 cells expressing a control or TRIM11 shRNA. **i**, **j** YFP-CL1 was expressed in control and Flag-TRIM11-expressing HCT116 cells. Cells were treated with cycloheximide (CHX) to inhibit new protein synthesis and analyzed by western blot at the indicated times post-treatment. Representative western blot (**i**) and relative YFP-CL1/tubulin ratios (**i**,** j**) are shown. In **i**, the amounts of lysates from control and TRIM11-expressing cells were adjusted to achieve similar levels of YFP-CL1 at 0 min. **k** Levels of YFP-CL1 when expressed alone or together with TRIM11 in HCT116 cells treated with control (DMSO) or MG132 (4 μM). **l** Steady-state levels of YFP-CL1 in HCT116 cells expressing a control or TRIM11 shRNA. In **e**–**h** and **j**, data represent the mean ± SEM (*n* = 3). **P < 0.01; ***P < 0.001. Uncropped blots are presented in Supplementary Fig. 9

To evaluate the role of TRIM11 in proteasomal degradation of misfolded proteins, we examined its effect on proteins that are conjugated with Lys48-linked polyubiquitin (K48 polyUb) chains, which constitute a major signal for proteasomal degradation^22^. Upon heat shock, K48 polyUb conjugates were dramatically increased in non-ionic detergent-insoluble (pellet or P), but not -soluble (supernatant or SN), lysates of human colon cancer HCT116 cells (Fig. 1a), suggesting that the insoluble K48 polyUb conjugates represented misfolded proteins that, upon the proteotoxic stress, were accumulated to levels exceeding the degradative capacity of the proteasome. We stably overexpressed TRIM11 in HCT116 cells (Supplementary Fig. 1a). This led to strong suppression of heat shock-induced increase in insoluble K48 polyUb conjugates (Fig. 1a). This effect of TRIM11 overexpression persisted during the recovery period when heat shocked cells were cultured at the physiological temperature (Fig. 1a). Conversely, we stably knocked down TRIM11 in HCT116 cells using two independent small hairpin RNAs (shRNAs). This strongly increased insoluble K48 polyUb conjugates even in unstressed cells (Fig. 1b). We observed similar effects of TRIM11 knockdown in human breast cancer MCF7 cells (Fig. 1c) and osteosarcoma U2OS cells (Fig. 1d).

To further investigate the effect of TRIM11 on overall misfolded proteins, we examined aggresomes, which are cellular inclusions that sequester misfolded proteins of amorphous structures^23^. Forced expression of TRIM11 reduced aggresomes in unstressed cells, and largely prevented their accumulation in heat shocked cells (Fig. 1e and Supplementary Fig. 1b). In contrast, silencing TRIM11 with shRNAs markedly increased aggresomes in unstressed cells (~100% increase; Fig. 1f). We also evaluated amyloid-like fibrils, which are highly ordered protein aggregates characterized by cross-β structures^24^. Forced expression of TRIM11 reduced amyloid-like fibrils in unstressed cells and near-completely prevented their increase in thermally stressed cells (Fig. 1g and Supplementary Fig. 1c), whereas silencing TRIM11 strongly elevated amyloid-like fibrils in unstressed cells (Fig. 1h).

To examine the effect of TRIM11 on individual misfolded proteins, we used a yellow fluorescent protein (YFP) fusion of the CL1 degron, which resembles a misfolded domain and commonly serves as a probe for UPS-mediated degradation^25,26^. Overexpressing TRIM11 accelerated the degradation of YFP-CL1 (Fig. 1i, j) and dramatically reduced its levels (Fig. 1k). Conversely, depleting TRIM11 noticeably increased YFP-CL1 (Fig. 1l). Together, these results indicate that TRIM11 promotes the degradation of misfolded proteins.

### TRIM11 enhances the degradation of normal regulatory proteins

**Fig. 2:**
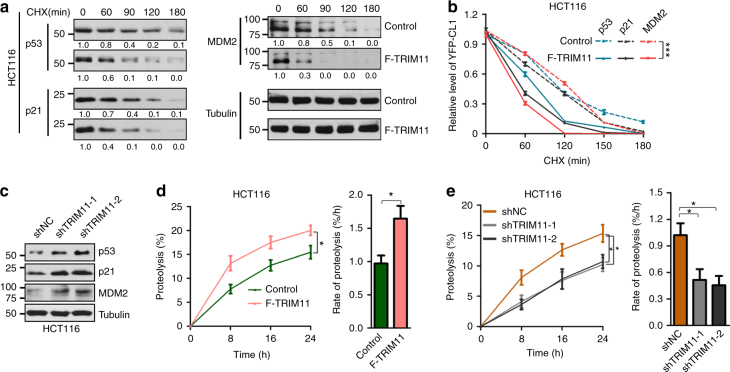
TRIM11 promotes the degradation of regulatory proteins and enhances overall rate of proteolysis. **a**,** b** Half-life of p53, p21, and Mdm2 in control and F-TRIM11-expressing HCT116 cells, assayed by CHX chase assays. Representative western blot of YFP-CL1 (**a**) and the quantification based on tubulin (**a**,** b**) are shown. The exposures of the corresponding control and F-TRIM11-expressing blots were adjusted to give similar band intensity at time 0. **c** Levels of endogenous p53, p21, and Mdm2 proteins in HCT116 cells without and with TRIM11 knockdown. **d**, **e** Overall protein degradation in TRIM11 overexpression (**d**), TRIM knockdown (**e**), and the corresponding control HCT116 cells. Cells were cultured in medium with [^3^H]-Phe and then in medium with non-radiolabeled Phe. [^3^H]-Phe released into the non-radiolabeled medium at the indicated times was measured, which was plotted as a percentage of total radioactivity incorporated into cellular proteins (left). Rate of proteolysis was calculated from the linear slopes at 8 h (right). In **b**, **d**, and **e**, data represent the mean ± SEM (*n* = 3). *P < 0.05; ***P < 0.001. Uncropped blots are presented in Supplementary Fig. 10

Next, we investigated the effect of TRIM11 on the degradation of normal regulatory proteins. The tumor suppressor p53, its target p21, and its inhibitor Mdm2 are turned over by the proteasome^27–29^. Forced TRIM11 expression accelerated the degradation of all three proteins (Fig. 2a, b). Conversely, depletion of TRIM11 slowed down the degradation of these proteins as reflected in an increase in their steady-state levels (Fig. 2c). TRIM11 promoted p53 degradation despite the reduction in Mdm2, suggesting that the effect of TRIM11 on p53 predominates that of Mdm2. In contrast, neither overexpression nor depletion of TRIM11 significantly affected the levels of p53, p21, and Mdm2 messenger RNA (mRNA) (Supplementary Fig. 2a, b).

### TRIM11 stimulates overall rate of proteolysis

The effect of TRIM11 on both misfolded and normal regulatory proteins prompted us to evaluate its role in global protein degradation. We employed a pulse-chase assay in which cells are cultured in medium containing ^3^H-Phe for a prolonged period of time to label virtually all cellular proteins, and then in medium containing an excess of nonradioactive Phe to permit the tracking of ^3^H-Phe-labeled proteins under the condition where reincorporation of ^3^H-Phe released from degraded proteins was minimized^30^. Of note, the overall rate of protein degradation rose by more than 60% in HCT116 cells ectopically expressing TRIM11 (Fig. 2d), while it declined by more than half in cells devoid of TRIM11 (Fig. 2e). Thus, by stimulating the degradation of both aberrantly and correctly folded proteins, TRIM11 exerts a strong influence on global proteolysis.

### TRIM11 activates the proteasome

**Fig. 3:**
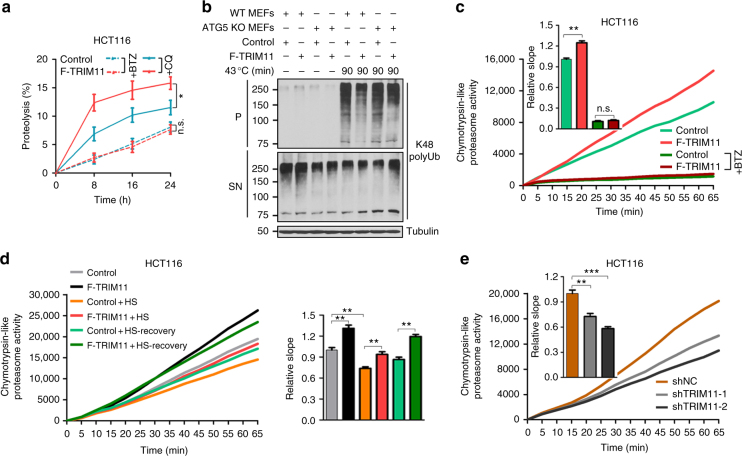
TRIM11 activates the proteasome. **a** Overall proteolysis in control and TRIM11-overexpressing HCT116 cells treated with BTZ (0.5 μM) or CQ (50 μM). **b** Levels of K48 polyUb conjugates in wild-type and *Atg5* KO MEF infected with vector or TRIM11 lentiviruses, and grown under normal or heat stress conditions. **c**, **d** Chymotrypsin-like proteasome activity in TRIM11-overexpressing HCT116 (**c**), TRIM11-depleted HCT116 cells (**d**), and the corresponding control cells, as measured by fluorometric substrate Suc-LLVY-AMC. In **c**, cells were also treated with BTZ (200 nM, 4 h). Slopes relative to that of control are shown, *n* = 7 (**c**, without BTZ), 6 (**c**, with BTZ), and 8 (**d**). **e** Chymotrypsin-like proteasome activity in control and TRIM11-overexpressing HCT116 cells cultured under unstressed, heat shock, and heat shock/recovery conditions. Slopes relative to that of control are shown, *n* = 7. In **a** and **c**–**e**, data represent the mean ± SEM (*n* = 3, unless otherwise indicated). *P < 0.05; **P < 0.01; ***P < 0.001. Uncropped blots are presented in Supplementary Fig. 10. n.s.: not significant

To assess whether the effect of TRIM11 is dependent on the proteasome, we used the proteasome inhibitors Z-Leu-Leu-Leu-al (MG132) and bortezomib (BTZ). When HCT116 cells were treated with MG132, TRIM11-mediated degradation of YFP-CL1 was effectively abrogated (Fig. 1k). Treatment with BTZ strongly reduced overall protein degradation (Fig. 3a), consistent with the notion that the UPS is the major cellular proteolytic mechanism. Under this condition, TRIM11 was no longer able to enhance overall protein degradation (Fig. 3a).

Autophagy is also implicated in protein turnover^31^. However, treatment of HCT116 cells with the autophagy inhibitor chloroquine (CQ) did not abrogate TRIM11-mediated enhancement of overall protein degradation (Fig. 3a). We also used mouse embryonic fibroblasts (MEFs) with homozygous knockout (KO) of *Atg5*, which encodes a critical autophagy regulator^31^. Upon heat shock, insoluble K48 polyUb conjugates were increased to a comparable level in wild-type and *Atg5* KO MEFs (Fig. 3b and Supplementary Fig. 3a). Moreover, when TRIM11 was ectopically expressed in these MEFs (Supplementary Fig. 3b), it prevented the accumulation of insoluble K48 polyUb conjugates to a similar extent (Fig. 3b). Therefore, we conclude that TRIM11 enhances protein degradation by the proteasome^21^.

The potent ability of TRIM11 to promote proteasomal degradation of both misfolded and correctly folded proteins prompted us to investigate whether TRIM11 may regulate a common step. The efficiency and specificity of ubiquitination is largely determined by ubiquitin E3s, which bind to both E2s and substrate proteins^2^, and ubiquitin E3 ligase activity has been shown for some TRIM proteins^17^. However, TRIM11 did not promote the ubiquitination of YFP-CL1 or p53 (Supplementary Fig. 3c, d).

We then tested the possibility that TRIM11 might activate the proteasome. Interestingly, using fluorogenic peptide substrates, we found that forced TRIM11 expression noticeably elevated all three proteasome activities (chymotrypsin-, caspase-, and trypsin-like) in HCT116 cells (~30% increase; Fig. 3c and Supplementary Fig. 3e, f), as well as chymotrypsin-like proteasome activity in MCF7 and U2OS cells (~20% increase; Supplementary Fig. 3g, h). TRIM11 also effectively prevented the decline in proteasomal peptidase activity in thermally stressed cells (Fig. 3d). Conversely, silencing TRIM11 led to a noticeable reduction in chymotrypsin-like proteasome activity in HCT116, MCF7, and U2OS cells (25-40% reduction; Fig. 3e and Supplementary Fig. 3i, j). The specificity of the fluorogenic peptide assay was underscored by use of the proteasome inhibitors BTZ and carfilzomib (CFZ), which effectively quenched the detected activities in both control and TRIM11-overexpressing cells (Fig. 3c and Supplementary Fig. 3e, f).

### TRIM11 associates with the proteasome

**Fig. 4:**
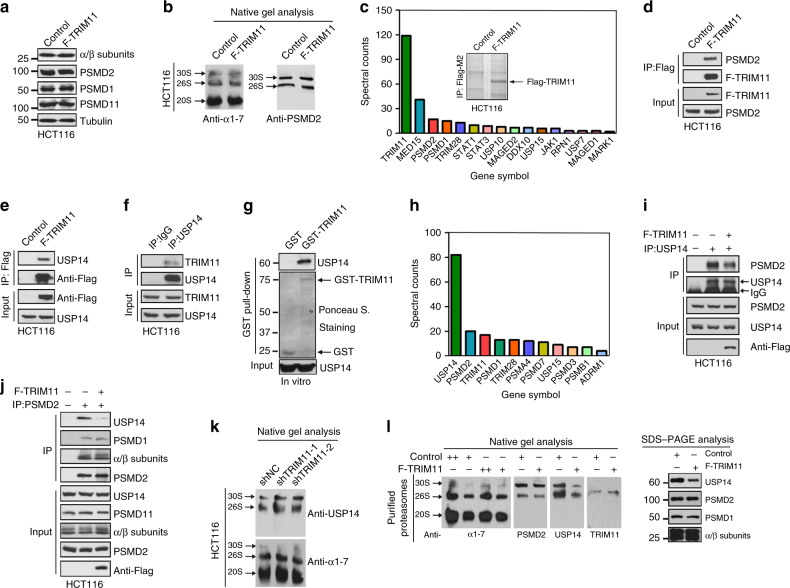
TRIM11 binds to both PSMD2 and USP14 and inhibits their interaction. **a** Control and Flag-TRIM11-expressing HCT116 cells were analyzed by western blot with the indicated antibodies. **b** Lysate fractions enriched in proteasomes (pellets of 5 h, 100,000×*g* centrifugation) from control and Flag-TRIM11-expressing HCT116 cells were analyzed by native gel and immunoblotted with antibody against the 20 S subunit α1-7 (left) or the 19 S subunit PSMD2 (right). **c**, **h** Control, Flag-TRIM11-expressing (**c**), and Flag-USP14-expressing (**h**) HCT116 cell lysates were incubated with beads conjugated with anti-Flag antibodies. Immunoprecipitates (IPs) were analyzed by mass spectrometry. Shown are spectral counts of peptides derived from the indicated proteins relative to those in the control IPs (**c**, **h**) and Coomassie blue staining of IPs (**c**, inset). **d** Interaction of Flag-TRIM11 and endogenous PSMD2 in HCT116 cells analyzed by co-immunoprecipitation (co-IP) assay. **e**, **f** Interaction of Flag-TRIM11 (**e**) and endogenous TRIM11 (**f**) with endogenous USP14 in HCT116 cells. **g** Interaction of USP14 with TRIM11 was analyzed by an in vitro pull-down assay using purified GST or GST-TRIM11 immobilized on beads (1 μg) and Flag-USP14 (2 μg). **i**,** j** Interaction of endogenous USP14 and PSMD2 in control and TRIM11-overexpressing cells was analyzed by co-IP assays using anti-USP14 (**i**) or anti-PSMD2 (**j**) antibody. **k** Cell Lysate fractions enriched with the proteasomes were isolated from control and TRIM11 knockdown HCT116 cells and analyzed by native gel electrophoresis and immunoblotting using α1-7 and USP14 antibodies. **l** Proteasomes purified from control and TRIM11-overexpressing HCT116 cells (++, 1 μg; +, 0.5 μg) were analyzed by native (left) and SDS (right) PAGE, followed by western blot with the indicated antibodies. Uncropped blots are presented in Supplementary Fig. 11

Next, we investigated the mechanism by which TRIM11 activates the proteasome. The synthesis of proteasome subunits is a predominant point of regulation^32^. However, TRIM11 did not significantly alter mRNA levels of any of the 20S and 19S subunits (Supplementary Fig. 4a, b). Nor did it affect the protein levels of several subunits that were tested, including the 20S subunits α and β, and the 19S subunits PSMD1, PSMD2, and PSMD11 (Fig. 4a). The proteasome can also be regulated at the level of assembly between the 20S CP and the 19S RP^32^. Nevertheless, control and TRIM11-overexpressing cells contained similar levels of free (20S), single-capped (26S), and double-capped (30S) CP particles, as shown by native gel electrophoresis (Fig. 4b).

There was a paucity of information on TRIM11 that might suggest its mechanism of action. Thus, we set out to identify novel TRIM11-interacting proteins using affinity purification. Lysates from control and Flag-TRIM11-expressing HCT116 cells were immunoprecipitated with anti-Flag antibody, and proteins in the immunoprecipitates were analyzed by mass spectrometry (MS). Several proteins were identified in Flag-TRIM11, but not the control, immunoprecipitates (Fig. 4c). Among them was MED15, a transcriptional regulator previously shown to bind to TRIM11 (ref. ^33^). Of note, PSMD1 and PSMD2, two subunits of the 19S proteasome, were also identified, and they were relative abundant in the Flag-TRIM11 immunoprecipitates based on the number of peptides detected by MS (Fig. 4c). We verified the interaction of TRIM11 with PSMD2 using a co-immunoprecipitation (co-IP) assay (Fig. 4d) and, as shown below, also detected the association of TRIM11 with the proteasome.

### TRIM11 interacts with USP14

PSMD1 and PSMD2 are the two largest subunits of the RP, serving as molecular scaffolds to recruit several proteasome-associated factors^3^. PSMD2, among others, interacts with the proteasome regulator USP14 (ref. ^6^). This, along with the PSMD2-TRIM11 interaction, raised the possibility that TRIM11 might also bind to USP14. Indeed, Flag-TRIM11 expressed in HCT116 cells interacted with endogenous USP14, based on a co-IP assay with anti-Flag antibody (Fig. 4e) and a reciprocal assay with anti-USP14 antibody (Supplementary Fig. 4c). Additionally, exogenous Flag-TRIM11 and HA-USP14 (Supplementary Fig. 4d, e), or endogenous TRIM11 and USP14 (Fig. 4f), also interacted with each other. The TRIM11-USP14 interaction is likely to be direct, as shown by an in vitro pull-down assay using purified recombinant proteins (Fig. 4g). Moreover, TRIM11 and USP14, whether exogenously or endogenously expressed, co-localized with each other in HCT116 cells (Supplementary Fig. 4f, g).

To understand the full complement of USP14-binding proteins, we performed another affinity purification using HCT116 cells stably expressing Flag-USP14. Several proteasome subunits—including the 19S subunits PSMD1, PSMD2, PSMD7, and PSMD3, and the 20S subunits PSMA4 and PSMB1—were identified in Flag-USP14, but not control, immunoprecipitates (Fig. 4h). Among them, PSMD2 appeared to be the most abundant, consistent with the previous finding that USP14 binds to the proteasome via PSMD2 (ref. ^6^). Interestingly, TRIM11 was also highly enriched in Flag-USP14 immunoprecipitates (Fig. 4h), supporting the notion that TRIM11 is a major non-proteasome protein that interacts with USP14.

### TRIM11 precludes the recruitment of USP14 to the proteasome

TRIM11 did not alter levels of the USP14 protein (Fig. 4e and Supplementary Fig. 4c–e). Given that USP14 reversibly associates with PSMD2 (refs. ^6,7^), we reasoned that TRIM11 might impede the USP14-PSMD2 interaction. Indeed, the association between endogenous USP14 and PSMD2 was strongly inhibited upon TRIM11 overexpression, as shown by reciprocal co-IP assays using anti-USP14 (Fig. 4i) and anti-PSMD2 (Fig. 4j) antibodies. In contrast, TRIM11 did not alter the association of PSMD2 with PSMD11 or the 20S core subunits α and β (Fig. 4j).

We also assessed the effect of TRIM11 on the recruitment of USP14 to the proteasome. By analyzing proteasome-enriched cell lysates from control and TRIM11-depleted HCT116 cells, we noted that the 20S and 26S proteasome contents were comparable in these cells, but the association of USP14 with 26S and 30S proteasomes was noticeably increased in TRIM11-depleted cells (Fig. 4k). Conversely, an analysis of affinity purified proteasomes from control and TRIM11-overexpressing cells showed that TRIM11 associated with the proteasome in control cells and this association increased in TRIM11-overexpressing cells (Fig. 4l), confirming the interaction of TRIM11 with the proteasome. Of note, the recruitment of USP14 to the proteasome was concomitantly reduced in TRIM11-overexpressing cells (Fig. 4l). Together, these results show that TRIM11 inhibits the association of USP14 with PSMD2, preventing it from docking on the proteasome.

In addition to TRIM11, TRIM28 was also identified as a potential USP14-interacting protein (Fig. 4h). Previously we showed that TRIM28 does not promote the degradation of misfolded proteins^21^. Consistently, silencing TRIM28 using two independent shRNAs (Supplementary Fig. 5a) failed to alter the levels of insoluble K48 polyUb conjugates (Supplementary Fig. 5b) or the USP14-PSMD2 interaction (Supplementary Fig. 5c), underscoring the specific effect of TRIM11 on the proteasome.

### TRIM11 suppresses USP14’s deubiquitinase activity

**Fig. 5:**
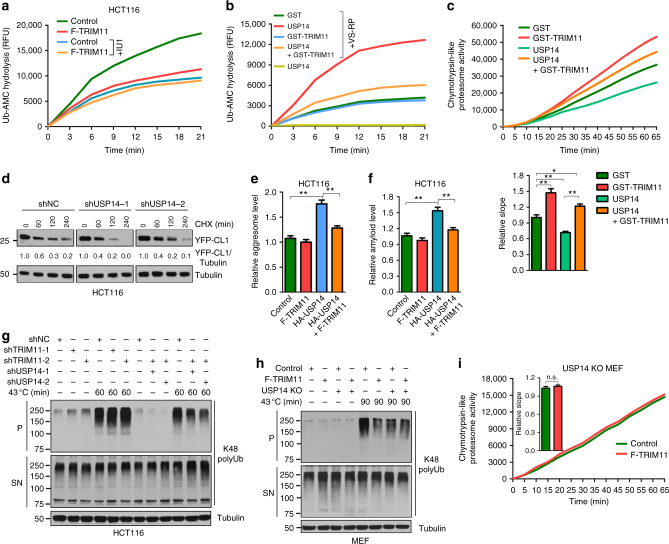
TRIM11 counteracts the effects of USP14 on proteasome activity and degradation of misfolded proteins. **a** Deubiquitinase activity in proteasome-enriched lysates from control and TRIM11-overexpressing HCT116 cells treated with vehicle (DMSO) or IU1 (100 μM). **b** RP pre-treated with Ub-VS (VS-RP, 5 nM) was incubated with recombinant GST, Flag-USP14, GST-TRIM11, or Flag-USP14 plus GST-TRIM11 proteins (50 nM each). Deubiquitinase activity was measured using Ub-AMC. **c** Chymotrypsin-like proteasome activity in HCT116 cell lysates incubated with the indicated recombinant proteins (50 nM each), was measured using Suc-LLVY-AM. Slopes relative to that of control are also shown, *n* = 7. **d** Turnover of YFP-CL1 in control and USP14-depleted HCT116 cells. The relative YFP-CL1/tubulin ratios were quantitated from three independent experiments. The exposures of the corresponding control and USP14-knockdown blots were adjusted to achieve comparable levels of YFP-CL1 at time 0. **e**,** f** Relative abundance of aggresome (**e**) and amyloid (**f**) in HCT116 cells expressing the indicated proteins. **g** K48 polyUb conjugates in HCT116 cells expressing the indicated shRNAs, and treated with and without heat shock. **h** Levels of K48 polyUb conjugates in wild-type and *Usp14* KO MEFs infected with control or TRIM11 lentiviruses, and grown under normal or heat stress conditions. **i** Chymotrypsin-like proteasome activity in control and TRIM11-overexpressing *Usp14* KO MEF cells, as measured by fluorometric substrate Suc-LLVY-AMC. Slopes relative to that of control are shown, *n* = 7. In **a**–**c**,** e**,** f**, and **i**, data represent the mean ± SEM (*n* = 3 unless otherwise indicated). *P < 0.05; **P < 0.01. Uncropped blots are presented in Supplementary Fig. 12. n.s.: not significant

USP14 has a minimal deubiquitinase activity in its free form, but is dramatically activated upon binding to the proteasome^7^. To evaluate the effect of TRIM11 on the deubiquitinase activity of USP14, we assayed deubiquitinase activity in proteasome-enriched cell lysates using ubiquitin-7-amido-4-methylcoumarin (Ub-AMC), a common substrate for deubiquitinating enzymes. Compared to the lysates from control HCT116 cells, the lysates from TRIM11-overexpressing HCT116 cells displayed a much reduced deubiquitinase activity (Fig. 5a). Treatment with the USP14 inhibitor IU1 (ref. ^11^) strongly reduced the deubiquitinase activity in the control lysates, and rendered TRIM11 ineffective in reducing deubiquitinase activity (Fig. 5a), indicating that TRIM11 specifically inhibits the deubiquitinase activity of USP14. To confirm this, we tested TRIM11 on recombinant USP14 protein that were activated by proteasome-enriched cell lysates or purified RPs. USP14 acquired strong deubiquitinase activity in the presence of proteasome-enriched cell lysates. However, this activity was effectively diminished by recombinant TRIM11 (Supplementary Fig. 5d, e). In the presence of RPs that were pre-treated with Ub-vinylsulfone (Ub-VS) to block the activity of the stably associated deubiquitinase (presumably UCH37)^11^, recombinant USP14 also gained a strong ability to hydrolyze Ub-AMC (Fig. 5b). Again, this proteasome-mediated USP14 activation was effectively prevented by recombinant TRIM11 (Fig. 5b). By removing ubiquitin chains via its deubiquitinase activity from proteasome-bound substrates, USP14 regenerates free ubiquitin and maintains its abundance in cells^10^. Forced TRIM11 expression reduced the levels of free ubiquitin (Supplementary Fig. 5f), likely due to the inhibition of USP14.

### TRIM11 relieves the inhibition of USP14 on the proteasome

As expected from the role of USP14 as a proteasome regulator^11,15^, USP14 knockdown increased (Supplementary Fig. 5g, h), while USP14 overexpression decreased (Supplementary Fig. 5h, i), proteasomal peptidase activity. However, the inhibitory effect of USP14 overexpression on proteasomal peptidase activity was effectively relieved by the co-expression of TRIM11 (Supplementary Fig. 5i). Likewise, recombinant USP14 inhibited proteasomal peptidase activity in cell lysates, but this effect was counteracted by recombinant TRIM11 (Fig. 5c). USP14 inhibited the degradation of YFP-CL1, as overexpression of USP14 increased YFP-CL1 abundance (Supplementary Fig. 5j), while silencing USP14 accelerated YFP-CL1 degradation (Fig. 5d). TRIM11 abrogated the inhibitory effect of USP14 overexpression on YFP-CL1 in both unstressed and heat-stressed cells (Supplementary Fig. 5j). Moreover, forced USP14 expression markedly increased aggresomes (Fig. 5e) and amyloid-like fibrils (Fig. 5f) in cells. Again, TRIM11 effectively neutralized these pro-aggregation functions of USP14 (Fig. 5e, f).

To further determine whether the effect of TRIM11 on global protein degradation and the proteasome is due to the inhibition of USP14, we used cells where USP14 was knocked down or deleted. Silencing TRIM11 substantially increased insoluble K48 polyUb conjugates in unstressed (Figs. 1b–d and 5g) and thermally stressed (Fig. 5g) control HCT116 cells. However, this effect was diminished in USP14-knockdown HCT116 cells (Fig. 5g and Supplementary Fig. 5k). Conversely, overexpressing TRIM11 in WT MEFs (Supplementary Fig. 5l) decreased heat shock-induced insoluble K48 polyUb conjugates (Fig. 5h) and activated proteasomal peptidase activity (Supplementary Fig. 5m). Yet, TRIM11 failed to decrease the ubiquitinated species and activate proteasome in *Usp14* KO MEFs (Fig. 5h, i). Together, these results indicate that TRIM11 bolsters the function of the proteasome mainly by relieving the inhibitory effect of USP14.

### Structural determinants of the TRIM11-USP14 interaction

**Fig. 6:**
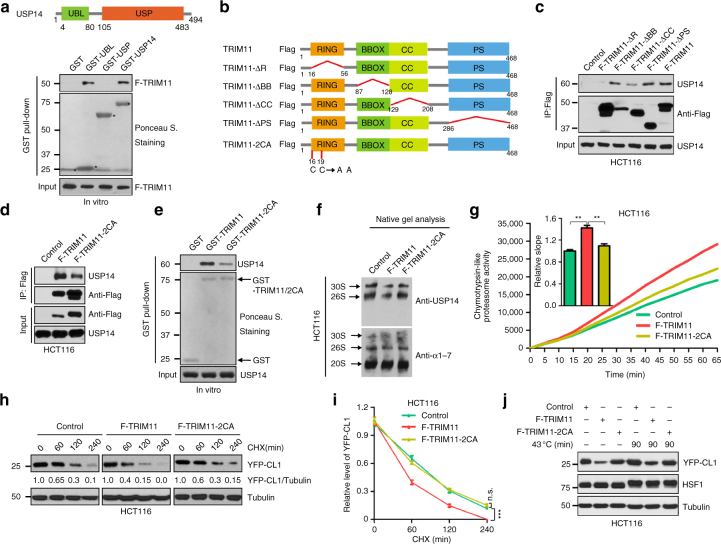
Structure determinants of the USP14-TRIM11 interaction. **a** Top: schematic diagram of USP14. The ubiquitin-like (UBL) and ubiquitin-specific protease (USP) domains, as well as the numbers of amino acids, are indicated. Bottom: interaction of the USP14 UBL domain with TRIM11. Beads-conjugated GST or GST-USP14 (2 μg each) was incubated with Flag-TRIM11 (1 μg). The input and beads-bound proteins were analyzed by western blot with anti-Flag antibody or Ponceau S staining. Asterisk indicates GST and GST fusions. **b** Schematic diagram of TRIM11 and its mutants. In TRIM11-2CA, the conserved Cys16 and Cys19 were mutated to Ala. Each construct was tagged with Flag epitope. **c**, **d** Interaction of Flag-TRIM11 proteins with endogenous USP14 in HCT116 cells was analyzed by co-IP assay. **e** Interaction of USP14 (2 μg) with GST, GST-TRIM11, and GST-TRIM11-2CA (1 μg each) was analyzed by in vitro pull-down assay. **f** Cell Lysate fractions enriched with the proteasomes were isolated from control, Flag-TRIM11-expressing, and Flag-TRIM11-2CA-expressing HCT116 cells, and analyzed by native gel electrophoresis and immunoblotting using α1-7 and USP14 antibodies. **g** Chymotrypsin-like proteasome activity in control, Flag-TRIM11-expressing, and Flag-TRIM11-2CA-expressing HCT116 cells. Slopes relative to that of vector are also shown (mean ± SEM, *n* = 8). **h**,** i** Half-life of YFP-CL1 in control, TRIM11-expressing, and TRIM11-2CA-expressing HCT116 cells. Representative western blots (**h**) and the relative YFP-CL1/tubulin ratios (**h**, **i**) are shown. The exposures of the corresponding control and TRIM11/TRIM11-2CA-expressing blots were adjusted to achieve comparable levels of YFP-CL1 at time 0. **j** Levels of YFP-CL1 in control, TRIM11-expressing, and TRIM11-2CA-expressing HCT116 cells grown under normal and heat stress conditions. In **g** and **i**, data represent the mean ± SEM (*n* = 3 unless otherwise indicated). **P < 0.01; ***P < 0.001. Uncropped blots are presented in Supplementary Fig. 13. n.s.: not significant

Next, we delineated the structural determinants of the TRIM11-USP14 interaction. USP14 contains an N-terminal UBL domain that binds to the RP, and a C-terminal USP domain for catalysis (Fig. 6a)^7^. We fused the UBL and USP domains individually to glutathione S-transferase (GST). In an in vitro pull-down assay, GST-UBL, but not GST-USP, bound to Flag-TRIM11 (Fig. 6a), indicating that the UBL domain mediates the interaction with TRIM11.

In addition to the N-terminal RBCC region, TRIM11 is composed of a C-terminal PRY-SPRY (PS) motif. To assess the domain(s) of TRIM11 that mediates the interaction with USP14, we constructed TRIM11 deletion mutants lacking each of the individual domains within the RBCC region (ΔR, ΔBB, or ΔCC), or the entire PS region (ΔPS) (Fig. 6b). TRIM11-ΔBB and -ΔPS retained the ability to interact with endogenous USP14 as shown by a co-IP assay (Fig. 6c). However, TRIM11-ΔCC partially, and TRIM11-ΔR completely, lost this ability (Fig. 6c). To further examine the role of the RING domain, we used a mutant in which two conserved Cys resides involved in Zn^2+^ binding (Cys16 and Cys19) were changed to Ala (TRIM11-2CA) (Fig. 6b and Supplementary Fig. 6a)^21^. TRIM11-2CA displayed a reduced interaction with USP14 both in cells (Fig. 6d) and in vitro (Fig. 6e). These results, especially the in vitro assay, indicate that the structure, rather than the E3 activity, of TRIM11 RING domain is required for interaction with USP14.

Endogenous USP14 mainly localized in the cytosol (Supplementary Fig. 4g). TRIM11-ΔBB and -ΔPS, like wild-type TRIM11, showed a similar localization pattern to USP14 (Supplementary Fig. 6b). In contrast, TRIM11-ΔR and TRIM11-2CA were mainly present in the nucleus, and TRIM11-ΔCC in the nucleus as well as the cytoplasm (Supplementary Fig. 6b). Thus, the RING domain is also required for the co-localization of TRIM11 with USP14.

TRIM11-2CA exhibited a noticeably reduced ability to displace USP14 from the proteasome (Fig. 6f) and to stimulate proteasomal peptidase activity (Fig. 6g). It also lost the ability to promote YFP-CL1 degradation in unstressed (Fig. 6h–j) and heat-stressed (Fig. 6j) cells. Collectively, these results suggest that TRIM11 regulates USP14 via a direct protein–protein interaction that involves the UBL domain of USP14 and the RING domain of TRIM11.

### TRIM11 enhances cell survival and proliferation

**Fig. 7:**
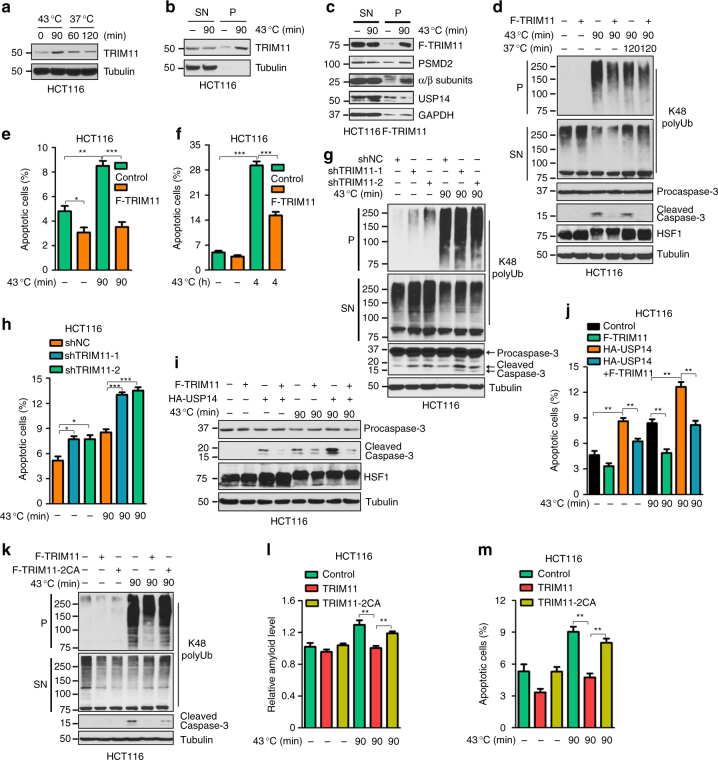
Role of the TRIM11-USP14 axis in cell growth and apoptosis. **a** Levels of endogenous TRIM11 in HCT116 cells grown at 43 °C for 0 or 90 min, or at 43 °C for 90 min, and then at 37 °C for 60 or 120 min. **b**, **c** Levels of endogenous TRIM11 (**b**), or Flag-TRIM11, the indicated proteasome subunits, and USP14 (**c**) in the SN and P fractions of HCT116 cells with and without heat shock at 43 °C for 90 min. **d**,** g** TRIM11 overexpression (**d**) or knockdown (**g**) HCT116 cells and the corresponding control cells grown under unstressed, heat shock, and heat shock plus recovery conditions were analyzed for K48 polyUb conjugates and caspase-3 activation. **e**, **f**,** h** Apoptosis in TRIM11 overexpression (**e**,** f**) or knockdown (**h**) HCT116 cells, and the corresponding control HCT116 cells, treated with and without heat shock. **i**,** j** Caspase-3 activation (**i**) and apoptosis (**j**) in HCT116 cells expressing the indicated proteins and grown under normal or heat stress conditions. **k**–**m** K48 polyUb conjugates, caspase-3 activation (**k**), amyloid-like fibrils (**l**), and apoptosis (**m**) in HCT116 cells expressing the indicated proteins and grown under normal and heat stress conditions. In **e**,** f**, **h**, **j**, **l**, and **m**, data represent mean ± SEM (*n* = 3 unless otherwise indicated). *P < 0.05; **P < 0.01; ***P < 0.001. Uncropped blots are presented in Supplementary Fig. 14

To investigate the role of the TRIM11-USP14 pathway in maintaining protein homeostasis, we examined the expression of TRIM11 under proteotoxic stress conditions. The levels of TRIM11 protein substantially increased when cells were heat shocked at 43 °C, and subsequently declined during a recovery period at 37 °C (Fig. 7a). The increase in TRIM11 occurred almost exclusively in the detergent-insoluble pellets (Fig. 7b, c), coincident with the enrichment of 20S subunits α and β as well as the 19S subunit PSMD2 in the same fraction (Fig. 7c). This was presumably due to the co-localization of proteasomes and the associated TRIM11 with misfolded proteins. In contrast, the amounts of USP14 were decreased in the pellets and conversely increased in the detergent-soluble SN (Fig. 7c), consistent with its displacement from the proteasome by TRIM11.

Heat stress altered cell morphology (Supplementary Fig. 7a), activated caspase-3 (Fig. 7d and Supplementary Fig. 7b), and induced apoptosis (Fig. 7e, f and Supplementary Fig. 7c). Overexpressing TRIM11 suppressed heat-induced morphology changes in HCT116 cells (Supplementary Fig. 7a). It also reduced caspase-3 activation in HCT116, MCF7, and U2OS cells correlating with a reduction in misfolded proteins (Fig. 7d and Supplementary Fig. 7b). Additionally, TRIM11 inhibited heat-induced apoptosis in HCT116 cells (Fig. 7e). Even under a severe heat stress condition when a substantial number of control cells underwent apoptosis (~30%), TRIM11 afforded cells a noticeable survival advantage, reducing apoptosis by half (Fig. 7f and Supplementary Fig. 7c). Conversely, depleting TRIM11 impaired growth of unstressed HCT116 cells (Supplementary Fig. 7d), and enhanced caspase-3 activation (Fig. 7g) and apoptosis (Fig. 7h) in both unstressed and stressed cells. Thus, TRIM11 enhances cell survival and proliferation.

### TRIM11 counteracts the effect of USP14 on cell growth

Consistent with the role of USP14 in suppressing the proteasome, depleting USP14 reduced caspase-3 activation (Supplementary Fig. 7e) and apoptosis (Supplementary Fig. 7f) in heat-stressed cells. Conversely, overexpressing USP14 impaired proliferation of unstressed cells (Supplementary Fig. 7g), and reduced cell attachment to plates (Supplementary Fig. 7h), promoted caspase-3 activation (Fig. 7i), and increased apoptosis (Fig. 7j) in both unstressed and stressed cells. Of note, TRIM11 counteracted the effects of USP14 overexpression, restoring cell attachment and proliferation (Supplementary Fig. 7g, h), decreasing caspase-3 activation (Fig. 7i), and enhancing cell viability (Fig. 7j). Compared to TRIM11, TRIM11-2CA displayed a much weaker activity. It was less effective in preventing heat shock-induced accumulation of K48 polyUb conjugates (Fig. 7k) and amyloid-like fibrils (Fig. 7l), reducing caspase-3 activation (Fig. 7k), and lessening apoptosis (Fig. 7m). Together, these results demonstrate that TRIM11 enhances cell survival and proliferation and suppresses apoptosis by inhibiting USP14.

### Opposing roles of TRIM11 and USP14 in tumorigenesis

**Fig. 8:**
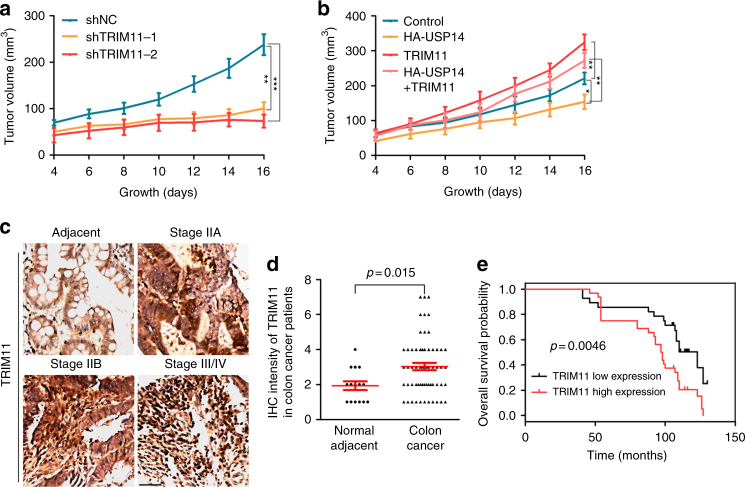
Opposing roles of TRIM11 and USP14 in tumorigenesis. **a**, **b** HCT116 cells stably expressing the indicated shRNAs (**a**) or proteins (**b**) were subcutaneously injected into nude mice. Shown are average tumor volumes over time (mean ± SEM, *n* = 4). *P < 0.05; **P < 0.01; ***P < 0.001. **c**, **d** Representative pictures of IHC (**c**) and statistical data (**d**) of TRIM11 expression in different stages of clinical colon cancer and adjacent normal tissues. Scale bar, 50 μm. *P* value was assessed using two-tailed Student’s *t* tests. **e** Kaplan–Meier analysis of cumulative survival probability of colon cancer subdivided by TRIM11 expression. The statistical significance (*P* = 0.0046) was assessed using log-rank test according to colon cancer patients with low or high expression of TRIM11. TRIM11 low expression, *n* = 28; TRIM11 high expression, *n* = 32

Enhanced degradation of misfolded proteins, attributable in part to the upregulation of TRIMs, promotes the initiation and maintenance of tumorigenic phenotypes^21^. To examine the role of TRIM11 in tumor cells, we implanted HCT116 cells with TRIM11 knockdown or overexpression in immunodeficient mice. Compared to control cells, TRIM11 knockdown cells displayed markedly reduced ability to form tumors in animals (Fig. 8a and Supplementary Fig. 8a), whereas TRIM11 overexpression cells showed a strongly enhanced ability (Fig. 8b and Supplementary Fig. 8b). To investigate the role of USP14 in tumorigenesis and its influence by TRIM11, we overexpressed USP14 alone or together with TRIM11. Like TRIM11 knockdown cells, USP14 overexpression cells formed substantially smaller tumors (Fig. 8b and Supplementary Fig. 8b). However, co-expression of TRIM11 effectively restored tumor growth (Fig. 8b and Supplementary Fig. 8b).

To further examine the role of TRIM11, we evaluated its expression in colon cancer tissues by immunohistochemistry (IHC). Interestingly, colon carcinoma tissues expressed TRIM11 at significantly higher levels compared to the normal adjacent tissues (Fig. 8c, d, and Supplementary Fig. 8c). An analyzing of clinical survival data according to TRIM11 expression levels revealed that high TRIM11 expression correlated with a significantly lower survival probability in colon cancer patients (Fig. 8e). Thus, TRIM11 contributes to tumor progression and may be an important clinical predictor for the survival of colon cancer patients.

## Discussion

With a remarkable array of substrates, the proteasome plays a crucial role in controlling protein homeostasis and various other cellular processes^4,5^. Emerging evidence indicates that the proteasome itself is subject to intricate regulation to meet the fluctuating cellular demand for proteolysis^32^. While the regulation can occur at the levels of expression and post-translational modification of proteasome subunits, a major mechanism is the association of the proteasome with factors that act directly on proteasome-bound ubiquitin chains, especially USP14. USP14 removes ubiquitin chains from substrates before they are committed to degradation. As such, USP14 regulates the substrate specificity of the proteasome and multitude of its activity, and it may also serve as a timing device to set the duration during which a substrate can reside on the proteasome^9–11,13,14,34^. Additionally, USP14 allosterically inhibits multiple proteasome activities^15^.

The notion that USP14 may serve as a focal point for regulating proteasome activity was consistent with a recent report that the kinase Akt activates USP14 via phosphorylation^35^. The current study reveals a distinct proteasome-regulatory pathway based on stable protein–protein interaction, in which TRIM11 binds to USP14 precluding its recruitment to the RP subcomplex of the proteasome. TRIM11 is likely a major non-proteasome protein that stably associates with USP14, and its effect on the proteasome, which is highly potent, appears to be mainly due to the suppression of USP14. TRIM11 interacts with the UBL domain of USP14 and may physically block its access to the RP. By displacing USP14, TRIM11 changes proteasome composition and suppresses both catalytic and non-catalytic effects of USP14. This likely prolongs the association of ubiquitinated proteins with the proteasome and also elevates the intrinsic proteolytic activity of the proteasome. Thus, TRIM11 broadens the range of proteasome substrates and increase its degradative power, augmenting global protein turnover.

TRIM proteins form a large family in metazoans with over 70 members in mice or humans^16,17^. Many TRIMs are shown to be ubiquitin E3 ligases that target various normal cellular proteins for degradation^18^. At least several TRIMs also possess SUMO E3 ligase activity^19^, which, in the case of PML, is involved in targeting misfolded proteins for degradation^20^. The current study reveals a remarkable function of TRIMs in the activation of the proteasome. It remains to be determined whether some other TRIMs act similarly to TRIM11, although TRIM28 does not appear to possess this ability. Still, it is notable that members of the TRIM family can promote substrate ubiquitination either directly (via ubiquitin E3 activity) or indirectly (via SUMO E3 activity) while bolstering proteasome activity. Thus, TRIM family likely plays a critical role in the coordination of the two fundamental activities of the UPS, matching the degradative capacity of the proteasome with the extent of substrate ubiquitination.

Maintenance of protein homeostasis is crucial for cell survival^36^. A role for TRIM11 in unstressed cells is showed by the noticeable retardation of cell proliferation upon its downregulation, accompanied by the accumulation of misfolded proteins. The expression of many components involved in protein homeostasis is frequently increased in response to proteotoxic stresses^37^. The expression of TRIM11 is upregulated upon heat shock, which likely represents a mechanism that adapts proteasome activity in the face of challenges to proteome integrity. Therefore, the TRIM11-USP14 axis may be an integral component of the protein homeostasis network, important in both unstressed and stressed cells.

The proteasome activity is often elevated in tumor cells^21,38,39^. Proteasome substrates include, among others, tumor suppressors (e.g., p53) and cell cycle inhibitors (e.g., p21 and p27). However, as many oncogenes (e.g., c-Myc) and cell cycle activators (e.g., cyclins) are also turned over by the proteasome, whether and how the increased degradation of regulatory proteins may be conducive to oncogenic growth remains unclear. We recently showed that enhanced degradation of misfolded proteins affords tumor cells strong anti-oxidant defense, and promotes the initiation and maintenance of malignant transformation^21^. This may provide an explanation for the role of the proteasome in oncogenesis. TRIM11 strongly promotes tumor growth. It also effectively abrogates the tumor-inhibitory effect of USP14 (Fig. 8). The current and previous^17,21^ studies indicate that the expression of TRIM11 and other TRIM proteins are increased in tumors. Thus, tumor cells may incorporate the TRIM11-USP14 axis to enhance proteasome function.

In contrast, impairment of the proteasome and accumulation of misfolded proteins are associated with neurodegenerative diseases^40^. TRIM proteins such as PML promotes the clearance of neurodegeneration-related misfolded proteins^20,21^. Deficiency in PML/TRIM19 exacerbates neurodegeneration in a mouse model of spinocerebellar ataxin 1 (SCA1), a progressive and lethal disease caused by a misfolded protein with an expanded polyglutamine stretch^20^. Among TRIM proteins, TRIM11 displays an especially strong activity^21^. Thus, properly adjusting the levels of TRIM proteins in general and TRIM11 in particular may be beneficial in treating both cancer and neurodegeneration.

## Methods

### Plasmids

Lentiviral vectors expressing TRIM11 were constructed into pTRPE-GFP-T2A- mCherry (kindly provided by J.L. Riley, University of Pennsylvania). Retroviral vectors expressing TRIM11 and TRIM11 mutants were constructed in pBabe-puro. GST-TRIM11, GST-TRIM11-2CA, GST-USP14, GST-UBL, GST-USP were constructed in pGEX expression vector. HA-Ubiquitin and p53 were constructed into pRK5 and pEGFP-C2, respectively. The following shRNA-expressing lentiviral plasmids were made in pLKO.1-puro and purchased from Sigma, with the clone numbers indicated: TRIM11 (TRCN0000033959 and TRCN0000033960), TRIM28 (TRCN0000017998 and TRCN0000017999), and USP14 shRNAs (TRCN000000742 and TRCN0000007426). Flag-HA-USP14 (human) was a gift from W. Harper (Addgene plasmid # 22569)^41^, and YFP-CL1, a gift from N. Dantuma (Addgene plasmid # 11950)^42^.

### Reagents

Primary antibodies against the following epitope or proteins were purchased from the indicated sources: tubulin (T6074, 1:2000), Flag M2 (F3165, 1:50), and Flag M2 magnetic beads (Sigma, 1:50); HA (71-5500, 1:1000) and HA agarose (Thermo Fisher Scientific, 1:50); GFP (M048-3, 1:1000) (MBL); GAPDH (NB300-221, 1:3000) (Novus Biologicals); TRIM11 (ABC926, 1:500) (EMD Millipore); 20S proteasome α/β subunits (BML-PW8155, 1:1000) and 20S proteasome α1–7 (BML-PW8195, 1:1000) (Enzo Life Sciences); p53 (DO-1, sc-126, 1:2000), p21 (sc-6246, 1:500), Mdm2 (sc-965, 1:500), ubiquitin (P4D1, sc-8017, 1:2000), USP14 (6E6, sc-100630, 1:1000) and HSF1 (H-311, sc-9144, 1:2000) (Santa Cruz Biotechnology); caspase-3 (9662, 1:1000), cleaved caspase-3 (9661, 1:1000), ATG5 (2630, 1:1000), and K48-linked polyubiquitin chain (4289, 1:1000) (Cell Signaling Technology); and USP14 (A300-919A, 1:1000), TRIM28 (A300-275A, 1:2000), PSMD1 (A303-851A, 1:1000), PSMD2 (A303-853A, 1:1000), and PSMD11 (A302-750A, 1:500) (Bethyl Laboratories). Secondary antibodies conjugated to HRP (1:5000) and AlexaFluor 488/568 (1:2000) were purchased from Santa Cruz Biotech and Invitrogen, respectively.

The following reagents were obtained from the indicate sources: Bortezomib (B-1408) (LC Laboratories); Carfilzomib (CFZ) (BioVision); complete protease inhibitor cocktail (Roche); cycloheximide (CHX) and IU1 (Calbiochem); recombinant human USP14 (BML-UW9840) and ProteoStat^®^ aggresome dye (Enzo Life Sciences); GST-Sepharose™ 4B beads (GE Healthcare Life Sciences); protein A/G agarose (Thermo Fisher Scientific); 3× Flag peptide, glutathione, benzonase, Z-Leu-Leu-Leu-al (MG132), chloroquine (CQ), and thioflavin T (ThT) (Sigma).

### Cell culture and stable cell lines

HCT116, U2OS, MCF7, HEK293T, and MEF were purchased from ATCC. HCT116 and U2OS were cultured in McCoy’s 5A medium (Life Technologies), and MCF7, HEK293T, and MEF were cultured in DMEM (Life Technologies), both supplemented with 10% FBS (HyClone), at 37 °C with 5% CO_2._ For heat shock, HCT116 and MEF cells were grown at 43 °C, and MCF7 and U2OS cells at 45 °C.

Lentiviruses and retroviruses were produced as previously described. Cells were infected using these viruses in the presence of 8 μg ml^−1^ polybrene and selected with appropriate antibiotics to generate stable cell lines. *Atg5* KO MEFs was kindly provided by H.W. Virgin (Washington University) and S. Cherry (University of Pennsylvania). *Usp14* KO MEFs were kindly provided by D. Finley (Harvard Medical School).

### Quantitative real-time PCR

Total RNA was extracted with TRIzol (Invitrogen) and 1 μg RNA was reverse-transcribed by using the First Strand cDNA Synthesis Kit (Marligen Biosciences). Quantitative PCR (qPCR) was performed using SYBR Green PCR Master Mix in the 7900HT Fast Real-Time PCR System (Applied Biosystems). The qPCR primers are shown in Supplementary Table 1.

### Immunoblotting and immunoprecipitation

For cell lysate fractionation, cells were lysed in same amount (~120 μl) NP-40 lysis buffer (50 mM Tris-HCl, pH 8.8, 100 mM NaCl, 5 mM MgCl_2_, 1 mM NaF, 0.5% NP-40, 2 mM DTT, 250 IU ml^−1^ benzonase, 1 mM PMSF, and 1× complete protease inhibitor cocktail) for 30 min on ice. Cell lysates were centrifuged at 4 °C and 16,000×*g* for 15 min. The SN was collected. The pellet (P) was re-suspended in ~60 μl pellet buffer (20 mM Tris-HCl, pH 8.0, 15 mM MgCl_2_, 2 mM DTT, 250 IU ml^−1^ benzonase, and 1× complete protease inhibitor cocktail) for 30 min on ice. The protein concentrations in the SN fraction were measured by Bradford assay (Bio-Rad Labs). Approximately 25% SN fractions (normalized based on protein concentrations) and the same percentages of the corresponding P fractions were resolved on SDS-PAGE and analyzed by western blot. To analyze protein half-life, cells were treated with CHX (50 μg ml^−1^) for different durations followed by western blot analysis.

For co-IP, cells were lysed in NP-40 lysis buffer and the lysates were incubated with anti-Flag (M2) or anti-HA antibody-conjugated agarose beads at 4 °C for 4 h, or with antibodies (1:50) against endogenous proteins followed by Protein A/G agarose beads at 4 °C for 4 h. After extensive washing, these beads were boiled in loading buffer and were analyzed by western blot.

Denaturing co-IP was performed as following: cells were lysed in buffer containing SDS, boiled, and diluted 20-fold in NP-40 lysis buffer without SDS. After centrifuged, the SN was incubated with anti-HA antibody-conjugated agarose at 4 °C overnight. The beads were washed sequentially in the NP-40 lysis buffer containing additional 0, 0.5, and 1 M KCl, boiled in loading buffer, and were analyzed by western blot.

### Native gel electrophoresis

To prepare the proteasome-containing extraction, cells were first homogenized for 10 min using a 1 ml tissue grinder in homogenization buffer (50 mM Tris-HCl, pH 7.5, 2 mM ATP, 1 mM DTT, 5 mM MgCl_2_, and 10% glycerol) and further lysed by passing 15 times through a 27-gauge needle. Lysates were centrifuged at 1500×*g* and 4 °C for 10 min. The SN was ultra-centrifuged at 100,000×*g* for 5 h to pellet the proteasome. The pellet was dissolved in the homogenization buffer, and protein concentrations were measured by Bradford assay. Samples were prepared by mixing with 6× native loading buffer (375 mM Tris pH 7.5, 60% glycerol, 0.06% bromophenol blue).

Native gel electrophoresis was performed with modifications^43,44^. Samples were resolved on a 4% native polyacrylamide gel at 4 °C, first for 1 h at 80 V, and then for 3 h at 150 V, in native running buffer (90 mM Tris, pH 8.3, 80 mM boric acid, 0.1 mM EDTA, 1 mM DTT, 1 mM ATP, and 5 mM MgCl_2_). Proteins were transferred to nitrocellulose membrane in transfer buffer (25 mM Tris, 192 mM glycine, 20% methanol, 0.1% SDS) at 4 °C and 30 V for 16 h, and analyzed by immunoblotting.

### Overall protein degradation

Determination of global protein degradation was performed based on the established methods^31,45^. Briefly, cells were cultured in complete medium containing ^3^H-Phe (5 μCi ml^−1^) (PerkinElmer) at 37 °C for 24 h to label cellular proteins. Cells were washed extensively with medium containing a high concentration of unlabeled Phe (2 mM), and were subsequently cultured in the same medium to prevent reutilization of radiolabeled Phe that was released from degraded proteins, in the presence of vehicle (DMSO), BTZ (0.5 μM) or CQ (50 μM). At different times, medium samples were collected, and proteins were precipitated with trichloroacetic acid (TCA; final concentration 10%). Cells were dissolved in solubilization buffer (0.1 N NaOH, 0.1% sodium deoxycholate). Both the total radioactivity initially incorporated into cellular proteins and TCA-soluble radioactivity at different times, which reflected degradation of pre-labeled proteins, was determined by liquid scintillation counting. Proteolysis was calculated as the amount of acid-soluble radioactivity relative to the total initial cellular radioactivity.

### Mass spectrometry

HCT116 cells stably expressing control vector, Flag-TRIM11, and HA-Flag-USP14 were lysed in buffer containing 50 mM HEPES, pH 7.4, 150 mM NaCl, 0.5% NP-40, 0.5% Triton X-100, 1 mM EDTA, 2 mM DTT, and freshly added protease inhibitors. Cell lysates were incubated with anti-Flag mAb M2 beads at 4 °C for 4 h. After extensive washing, the beads were eluted with PBS containing 0.2 mg ml^−1^ 3xFLAG peptide. Elution samples were analyzed by MS at Quantitative Proteomics Resource Core of the University of Pennsylvania with the following methods. Desalted peptides were analyzed on a Q-Exactive (Thermo Scientific) attached to an EasyLC system run at 300 nl min^−1^. Peptides were eluted with a gradient from 2 to 32% acetonitrile (ACN) over 50 min and to 98% ACN over 10 min in 0.1% formic acid. Data dependent acquisition mode with a dynamic exclusion of 45 s was enabled. One full MS scan was collected with scan range of 500–2000 *m*/*z*, resolution of 70 K, maximum injection time of 50 ms, and automatic gain control (AGC) of 1e6. A series of MS2 scans were acquired for the most abundant ions from the MS1 scan (top 12). Ions were filtered with charge 2–4. An isolation window of 2.0 *m*/*z* was used with quadruple isolation mode. Ions were fragmented using higher-energy collisional dissociation with collision energy of 24%. Orbitrap detection was used with scan range of 200–2000 *m*/*z*, resolution of 17.5 K, maximum injection time of 200 ms, and AGC of 5e4. For peptide identification and quantification, MaxQuant version 1.5.3.30 was used to process the raw spectra^43^. The uniprot human database was used for database searching. Search parameters were used with the default setting, including precursor mass tolerance of 20 p.p.m., fragment mass tolerance of 20  p.p.m., trypsin cleavage, and up to 2 mis-cleavage. Carbamidomethyl [C] was set as fixed modification, while oxidation [M] was set as variable modifications. The target-decoy approach was used to filter the search results^44^, in which the false discovery rate was <1% at the peptide and protein level. MS was also performed at Taplin Mass Spectrometry Facility of Harvard Medical School.

### Protein purification and in vitro pull-down assay

Flag-TRIM11 and HA-Flag-USP14 were immunoprecipitated from HCT116 cells stably expressing these proteins using anti-Flag M2 beads. Beads were washed sequentially with NP-40 lysis buffers containing an additional 0, 0.5, and 1 M KCl, respectively. The Flag-tagged proteins were eluted in the PBS buffer containing 0.2 mg ml^−1^ 3xFLAG peptide.

GST, GST-TRIM11, GST-USP14, GST-UBL, and GST-USP were expressed in *Escherichia coli* BL21 (DE3) and purified as following: the bacteria were grown at 37 °C and protein expression was induced with 0.2 mM IPTG. GST proteins were purified with glutathione beads, and eluted with 20 mM glutathione in PBS buffer.

For in vitro pull-down assay, purified USP14 or Flag-TRIM11 was incubated with GST proteins bound to glutathione beads (1 or 2 μg each protein) at 4 °C for 4 h. The beads were washed four times with NP-40 lysis buffer, boiled in loading buffer, and analyzed by western blot.

### Proteasomes purification

Proteasomes were purified using the Rapid 26S Proteasome Purification Kit (Ubiquitin-Proteasome Biotechnologies)^45^. Cell lysates were collected, sonicated, and then centrifuged at 100,000×*g* for 1 h. SNs were incubated with GST-UBL (0.2 mg ml^−1^) and glutathione beads (200 μl) at 4 °C for 6 h. After washing, the GSH-sepharose-bound 26S proteasome was eluted by His-UIM (2 mg ml^−1^). To remove the His-UIM, the combined eluate was incubated with Ni-NTA agarose (120 μl) at 4 °C for 20 min. The SN contained purified 26S proteasomes were obtained after subsequent spinning out Ni-NTA-bound His-UIM. Proteasome concentration was measured by the Bradford assay.

### Measurement of aggresome and amyloid-like fibrils

Cells were fixed by 4% paraformaldehyde (PFA) and permeabilized by 0.5% Triton X-100. After washing with PBS, cells were incubated with ProteoStat^®^ aggresome dye (Enzo Life Sciences) or 10 mM ThT in PBS at room temperature (RT; 25 °C) for 30 min^21,46^. Fluorescence was measured by a BD Accuri™ C6 flow cytometer (BD Biosciences) and analyzed by FlowJo software.

### MTT assay

96-well plates were seeded with 2500 cells per well in triplicates and cultured in complete medium. Cells were stained with 3-(4,5-dimethylthiazol-2-yl)-2,5-diphenyltetrazolium bromide (MTT) (Promega) at the indicated time points. Viable cells were determined by measuring optical density (OD) at 490 nm.

### Apoptosis analysis

Apoptotic cells were detected by FITC Apoptosis Detection Kit I (BD Biosciences). A total of 1 × 10^6^ cells were re-suspended in 1 ml binding buffer and incubated with annexin V-FITC and propidium iodide for 15 min at RT. The apoptotic cells were measured by flow cytometry and analyzed by FlowJo software.

### Proteasome activity assay

The activity of the 26S proteasome was measured^47^ by following steps. Cells were lysed in a cytosolic extract buffer on ice and were centrifuged at 10,000×*g* for 15 min at 4 °C. Four to eight micrograms of total protein was diluted with 26S proteasome assay buffer in a 96-well microtiter plate (BD Falcon), and incubated with fluorogenic substrate. Suc-LLVY-AMC, Ac-nLPnLD-AMC, and Ac-RLR-AMC (Enzo) were used to measure chymotrypsin-, caspase-, and trypsin-like proteasome activity, respectively. Fluorescence released by AMC fluorescence was monitored on a microplate fluorometer (Infinite M200, Tecan) every 5 min at 37 °C for 1 h. The specificity of the assay was assessed using the proteins inhibitors bortezomib (BTZ, 0.2 μM) and carfilzomib (CFZ, 0.5 μM), the latter being an effective inhibitor for the trypsin- and caspase-like activity.

### Ub-AMC hydrolysis activity of USP14

19S proteasomes (PA700, Boston Biochem) were pre-treated with Ub-VS (ubiquitin vinyl sulfone, Boston Biochem), an inhibitor of deubiquitinating enzymes, at 30 °C for 2 h to block the associated deubiquitinating activity. USP14 (Enzo) was mixed with the pre-treated 19S proteasomes or proteasome-enriched cell lysates, along with other purified proteins as indicated, in Ub-AMC assay buffer (50 mM Tris-HCl, pH 7.5, 5 mM MgCl_2_, 1 mM EDTA, 1 mM ATP, 1 mM DTT, and 1 mg ml^−1^ BSA). The reaction was initiated upon the addition of Ub-AMC (Boston Biochem), and the fluorescence of released AMC was monitored on a microplate fluorometer (Infinite M200, Tecan), using 345 nm and 445 nm wavelengths of excitation and emission, every 3 min at 37 °C.

### Tumor xenografts

Cells (1 × 10^6^) were mixed with matrigel membrane (BD Biosciences) and were injected subcutaneously into the flanks of 4–6-week-old athymic Balb-c nu/nu male mice (Taconic Farms, Germantown, NY, USA). Tumor length and width were evaluated by vernier caliper every 2 days, and the tumor volume was calculated using the formula *π*/6 × (width)^2^ × length. Sixteen days post injection, the mice were killed, and the tumors were isolated and weighed. All animal experiments were performed in accordance with relevant guidelines and regulations and were approved by the University of Pennsylvania Institutional Animal Care and Use Committee (IACUC).

### Immunofluorescence and immunohistochemistry

For immunofluorescence, cells cultured on coverslips were washed with PBS, fixed with 4% PFA for 30 min, and permeabilized with 0.15% Triton X-100 for 15 min. After washing with PBS, cells were blocked with 3% BSA for 30 min, incubated with the indicated primary and secondary antibody overnight at 4 °C or 1 h at RT, respectively. Cells were mounted by medium containing DAPI (Vector Labs) and observed using a fluorescence microscope (Olympus).

For IHC and the following survival data analysis, we purchased the colon cancer tissue array (CO953) from US Biomax. The IHC staining by TRIM11 antibody was performed by Histology and Gene Expression Core at the University of Pennsylvania Perelman School of Medicine. After pictures were taken from IHC, relative intensity of TRIM11 was quantified using ImageJ. This was stratified into TRIM11 low- and high-expression groups according to the IHC intensity of TRIM11, and the percentage of survival probability was assessed according to the two groups and clinical survival data of colon cancer patients.

### Data analysis

All experiments were repeated at least three times. Data analysis was used with GraphPad Prism 5 software (GraphPad Software, USA) through the unpaired two-tailed Student’s *t* test or two-way analysis of variance analysis. The bands of western blot were quantified using ImageJ (National Institutes of Health).

### Data availability

All data supporting the findings in this study are available from the corresponding author upon reasonable request.

## Electronic supplementary material

Supplementary Information(PDF 4139 kb)
