# Supplementary material for: TRIM11 activates the proteasome and promotes overall protein degradation by regulating USP14
Source: Nat Commun. 2018 Mar 26;9:1223. doi: 10.1038/s41467-018-03499-z (PMC5964324; doi:10.1038/s41467-018-03499-z)

TRIM11 activates the proteasome and promotes overall protein degradation by regulating USP14

Chen *et al*.

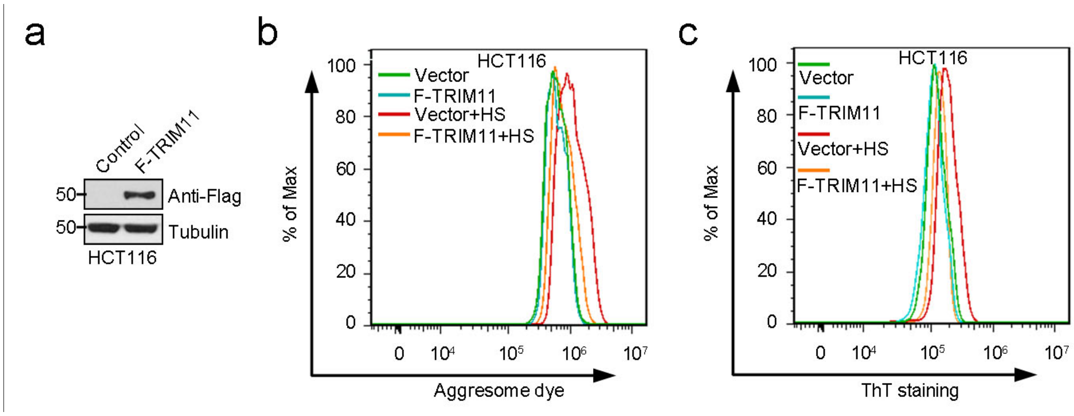

Supplementary Figure 1 | TRIM11 promotes the degradation of misfolded proteins.
(**a**) Western blot analysis of control and Flag-TRIM11-expressing HCT116 cells. (**b**, **c**) Flow cytometry analysis of aggresomes (**b**) and amyloid fibrils (**c**) using aggresomes dye and Thioflavin T (ThT), respectively. Related to Fig. 1e, g.

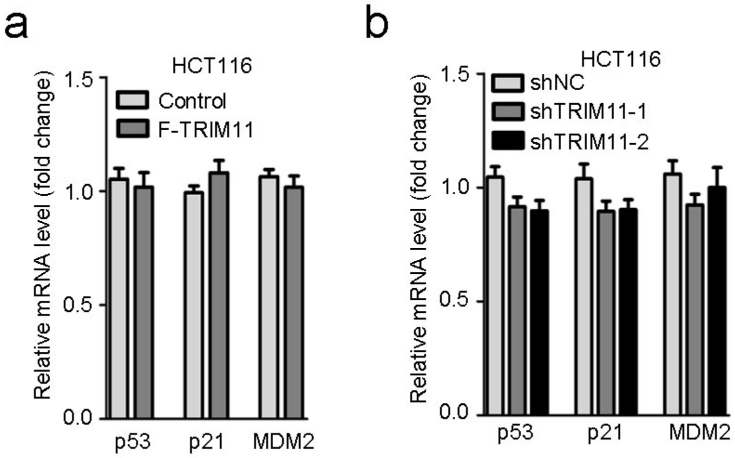

Supplementary Figure 2 | TRIM11 promotes the degradation of normal regulatory proteins. (**a**, **b**) Relative levels of p53, p21, and Mdm2 mRNAs in HCT116 cells with TRIM11 overexpression (**a**) or knockdown (**b**), and the corresponding control HCT116 cells, were measured by quantitative real-time PCR. Data are mean ± SEM, n = 3.

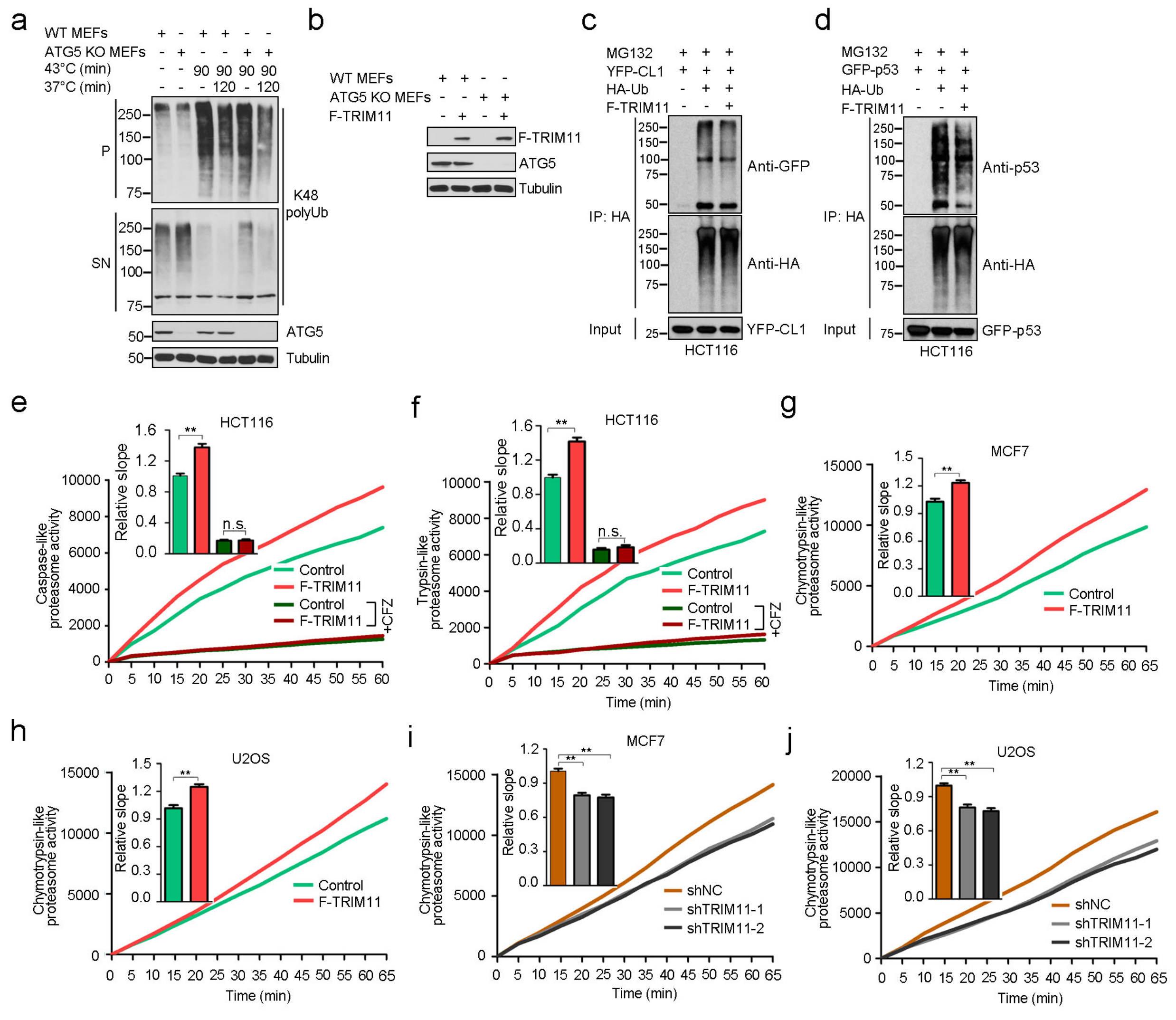

Supplementary Figure 3 | TRIM11 activates the proteasome

(**a**) Levels of K48 polyUb conjugates in wild type (WT) and *Atg5* KO MEFs grown under normal, heat stress, and heat stress plus recovery conditions. (**b**) Western blot analysis of WT and *Atg5* KO MEFs with and without Flag-TRIM11 expression. (**c**, **d**) Control and Flag-TRIM11-expressing HCT116 cells were transfected with HA-Ubiquitin (HA-Ub) plus YFP-CL1 (**c**) or p53-GFP (**d**). Cells were treated the proteasome inhibitor MG132. Extracts were denatured in SDS-containing buffer, diluted in non-denaturing buffer, and were incubated with anti-HA antibody. Immunoprecipitates and inputs were analyzed by western blot. (**e**, **f**) Caspase-like (**e**) and trypsin-like (**f**) proteasome activities in control and Flag-TRIM11-expressing HCT116 cells treated with or without carfilzomib (CFZ, 0.5 μM), measured by Ac-nLPnLD-AMC and Ac-RLR-AMC, respectively. Slopes relative to that of control are shown, n = 7. (**g**, **h**) Chymotrypsin-like proteasome activity in TRIM11-overexpressing MCF7 (**g**) and U2OS (**h**) cells and the corresponding control cells, measured by Suc-LLVY-AMC. Slopes relative to that of control are shown, n = 8. (**i**, **j**) Chymotrypsin-like proteasome activity in MCF7 (**i**) and U2OS (**j**) cells expressing control (shNC) or TRIM11 shRNA was measured by Suc-LLVY-AMC. Slopes relative to that of control are shown, n = 7. In **e**-**j**, data represent the mean ± SEM (n = 3 unless otherwise indicated). ** $P < 0.01$; n.s., not significant.

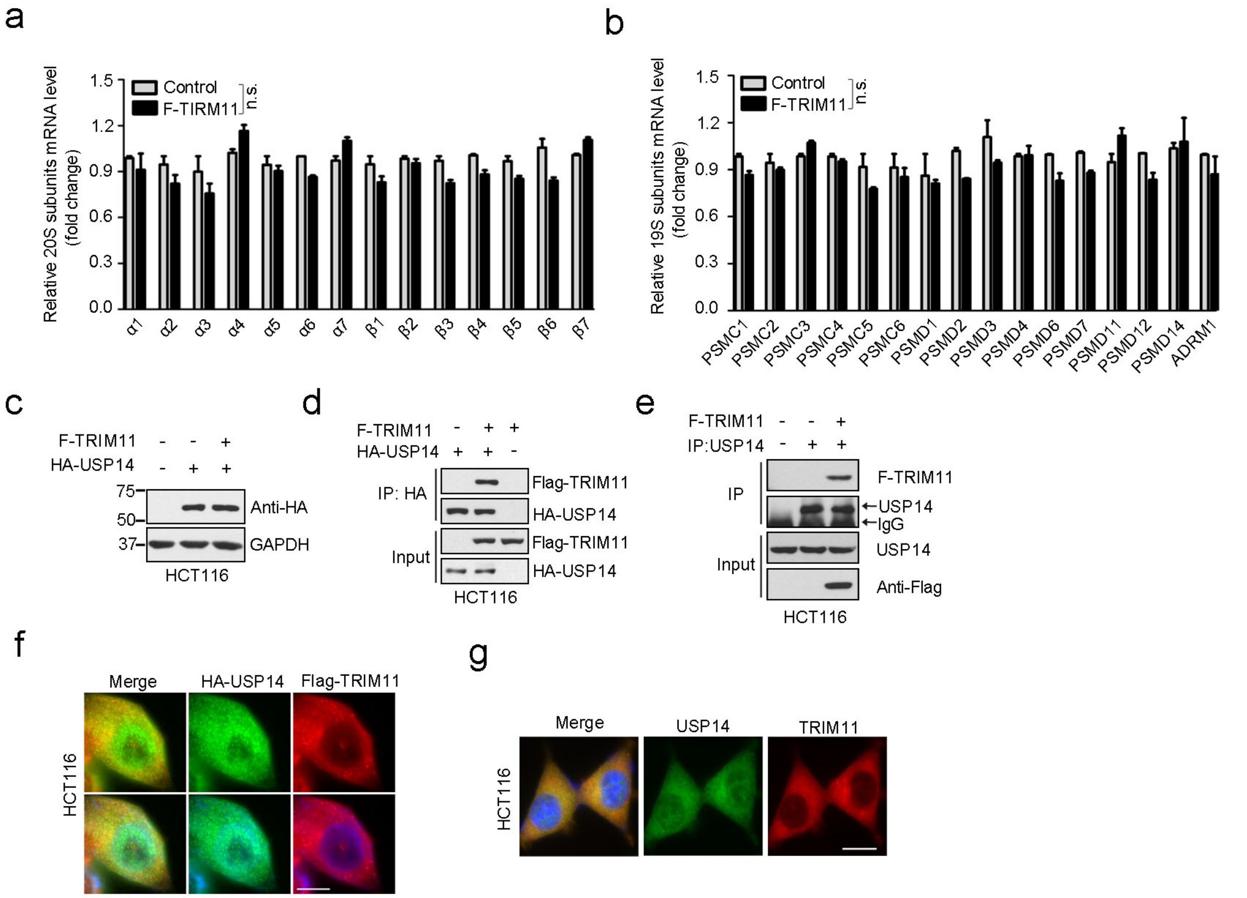

Supplementary Figure 4 | TRIM11 does not alter the expression of proteasome subunits and instead interacts with USP14

(**a**, **b**) Relative mRNA levels of proteasome subunits in HCT116 cells with and without TRIM11 overexpression. Data represent mean ± SEM, n = 3. Statistical significance was assessed using two-tailed Student's t-tests. n.s., not significant. (**c**) Interaction of Flag-TRIM11 with endogenous USP14 in HCT116 cells was assayed by co-IP assay. (**d, e**) Western blot analysis of HCT116 cells expressing Flag-TRIM11 and HA-USP14 as indicated (**d**) and co-IP assay of the Flag-TRIM11:HA-USP14 interaction (**e**). (**f**, **g**) Co-localization of Flag-TRIM11 and HA-USP14 (**f**), and co-localization of endogenous TRIM11 and USP14 (**g**), were analyzed by fluorescence microscopy. Scale bar, 10 μm.

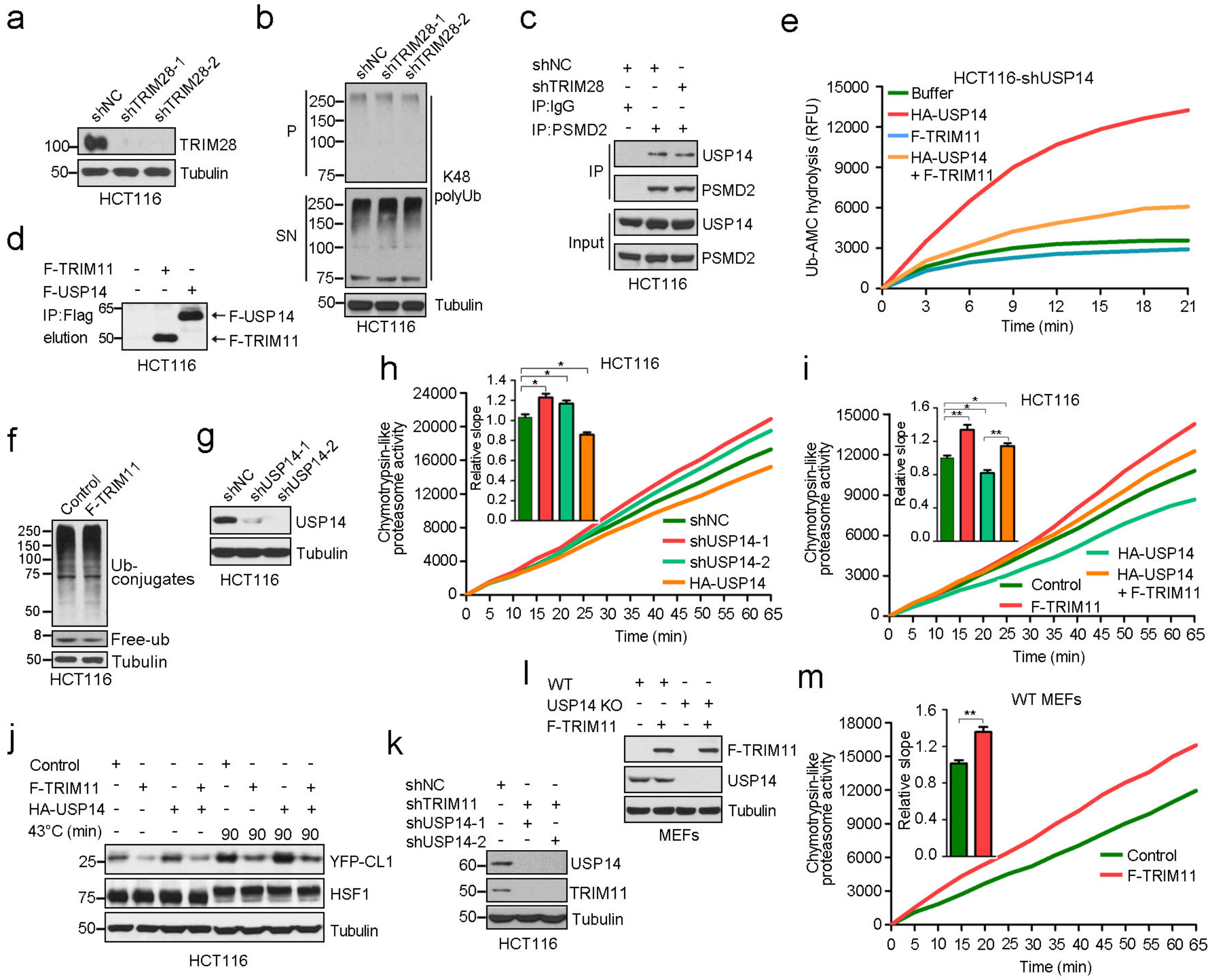

Supplementary Figure 5 | TRIM11 regulates the activity and function of USP14

(**a, b**) Levels of TRIM28 (**a**) and K48 polyUb conjugates (**b**) in HCT116 cells expressing control or TRIM28 shRNA. (**c**) Interaction of endogenous USP14 and PSMD2 in control and TRIM28-knockdown HCT116 cells. (**d**) Recombinant Flag-TRIM11 and Flag-USP14 (Flag-HA-USP14) proteins purified from HCT116 cells. (**e**) Hydrolysis of Ub-AMC by lysates from USP14-knockdown HCT116 cells, in the presence of the indicated proteins. (**f**) Levels of conjugated and free ubiquitin (Ub) in control and TRIM11-overexpressing cells. (**g**) Levels of USP14 in HCT116 cells expressing control and USP14 shRNAs. (**h)** Chymotrypsin-like proteasome activity in HCT116 cells expressing shNC, shUSP14-1, shUSP14-2, or HA-USP14 was measured by fluorometric substrate Suc-LLVY-AMC. Slopes relative to that of shNC are shown, n = 8. (**i**) Chymotrypsin-like proteasome activity in lysates of HCT116 cells expressing the indicated proteins was measured using Suc-LLVY-AM. Slopes relative to that of control are also shown, n = 8. (**j**) YFP-CL1 levels in HCT116 cells expressing the indicated proteins grown under normal and heat stress conditions. (**k**) Western blot analysis of HCT116 cells expressing the indicated shRNAs. (**l**) Western blot analysis of WT and *USP14* KO MEFs with and without Flag-TRIM11 expression. (**m**) Chymotrypsin-like proteasome activity in TRIM11-overexpressing MEF cells and the corresponding control cells, as measured by fluorometric substrate Suc-LLVY-AMC. Slopes relative to that of control are shown, n = 7. In **h**, **i** and **m**, data represent the mean ± SEM (n = 3). Statistical significance was assessed using two-tailed Student's t-tests. * P <0.05; ** P < 0.01

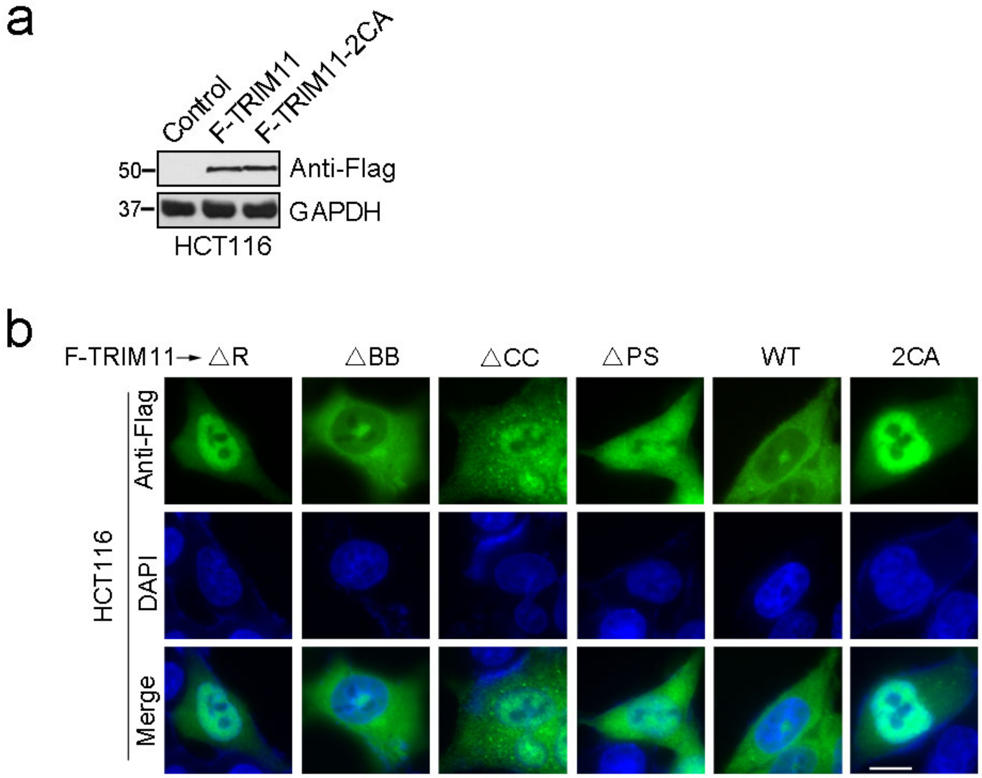

Supplementary Figure 6 | Cellular localization of wild type and mutant TRIM11 proteins
(**a**) Western blot analysis of HCT116 cells with and without Flag-TRIM11 or Flag-TRIM11-2CA expression. (**b**) Localization of wild type (WT) and mutant Flag-TRIM11 proteins in HCT116 cells, analyzed by fluorescence microscopy. TRIM11 was detected by anti-Flag antibody, and DNA by DAPI. Scale bar, 10 µm.

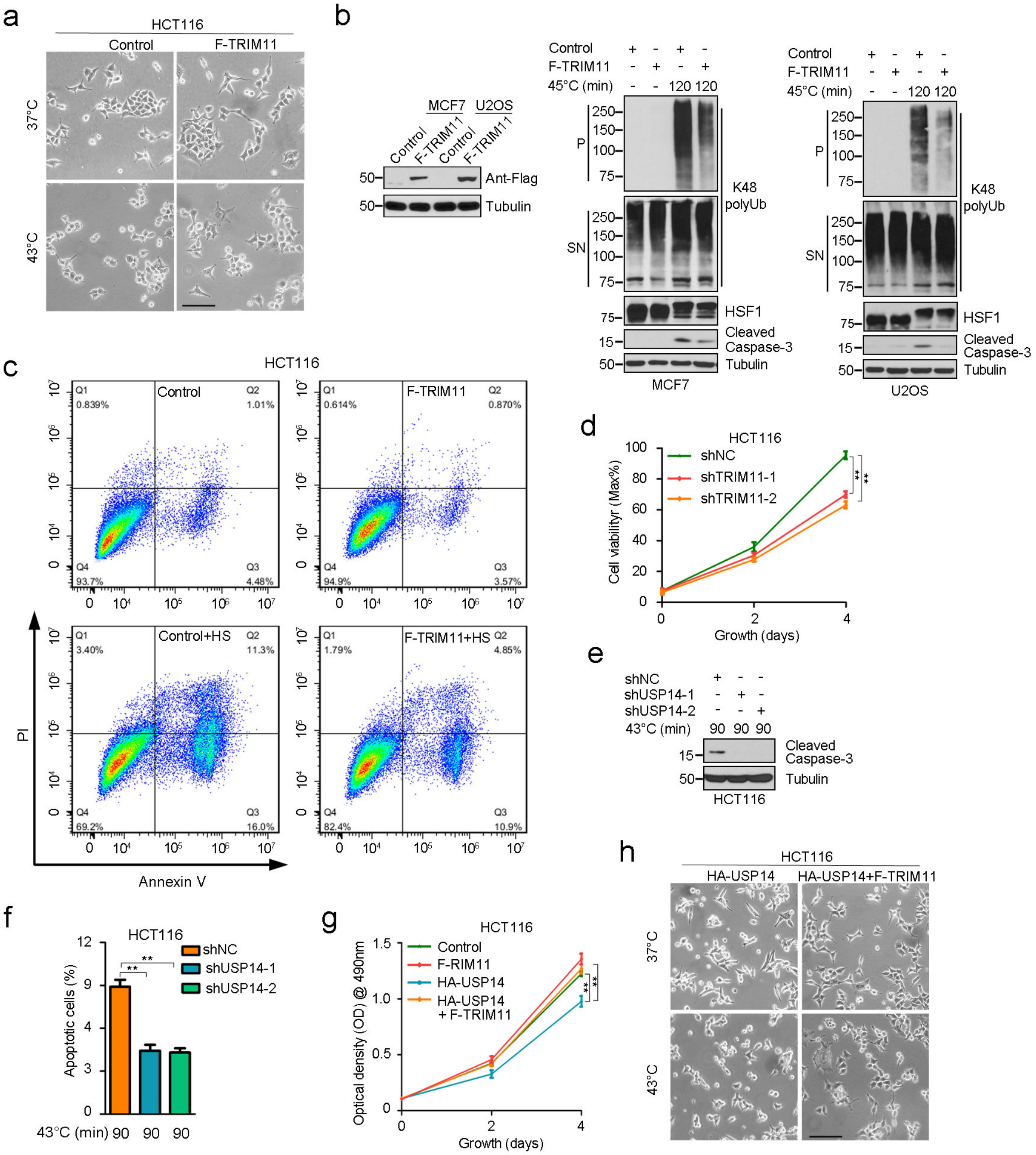

Supplementary Figure 7 | TRIM11 promotes cell survival and counteracts USP14
(**a**) Morphology of HCT116 cells stably expressing control vector or Flag-TRIM11 under normal and heat stress conditions. Scale bar, 50 µm. (**b**) Western blot analysis of MCF7 and U2OS cells stably with and without Flag-TRIM11 expression (left), and K48 polyUb conjugates and caspase-3 activation in MCF7 (middle) and U2OS (right) cells with and without Flag-TRIM11 expression and grown under normal and heat stress conditions. (**c**) Apoptosis in control and Flag-TRIM11-expressing HCT116 cells was assayed by annexin V and PI staining and flow cytometry, corresponding to Fig. 7f. Q1: PI-positive, Q2: annexin V- and PI-positive, Q3: annexin V-positive,

Q4: annexin V- and PI-negative. (**d**) Proliferation of control and TRIM11-knockdown HCT116 cells, measure by MTT assay. Data are relative optical density at 490 nM (mean ± SEM, n = 3). (**e**, **f**) Caspase-3 activation (**e**) and apoptosis (**f**) in control and USP14-depleted HCT116 cells grown under heat stress condition. (**g**) Proliferation of HCT116 cells expressing the indicated proteins was measured by MTT assay. (**h**) Morphology of HCT116 cells expressing the indicated proteins under normal and heat stress conditions. Scale bar, 50 μm. In **d**, **f** and **g**, data represent the mean ± SEM (n = 3). Statistical significance was assessed using two-tailed Student's t-tests. ** $P < 0.01$.

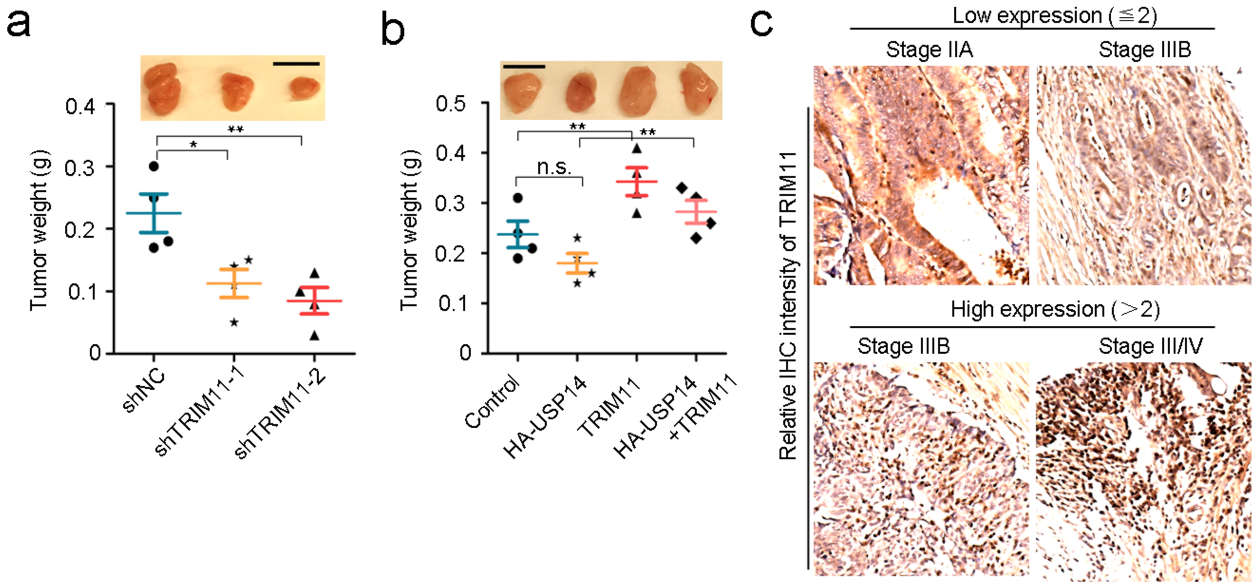

Supplementary Figure 8 | Role of TRIM11 and USP14 in tumor growth

(**a**, **b**) HCT116 cells expressing shNC, shTRIM11-1, or shTRIM11-2 (**a**), or the indicated proteins (**b**), were subcutaneously injected into nude mice. Shown are representative images of tumors, as well as average tumor weights at day 16 (n = 4). Scale bar, 1 cm. Data represent the mean ± SEM. Statistical significance was assessed using two-tailed Student's t-tests. * P <0.05; ** P < 0.01; n.s., not significant. (**c**) Representative IHC images of colon cancer samples with low and high TRIM11 expression, which are defined as relative TRIM11 intensity in the tumor versus adjacent normal tissue less than 2 and equal to or greater than 2, respectively.

Supplementary Figure 9 | Uncropped western blot images related to Figure 1

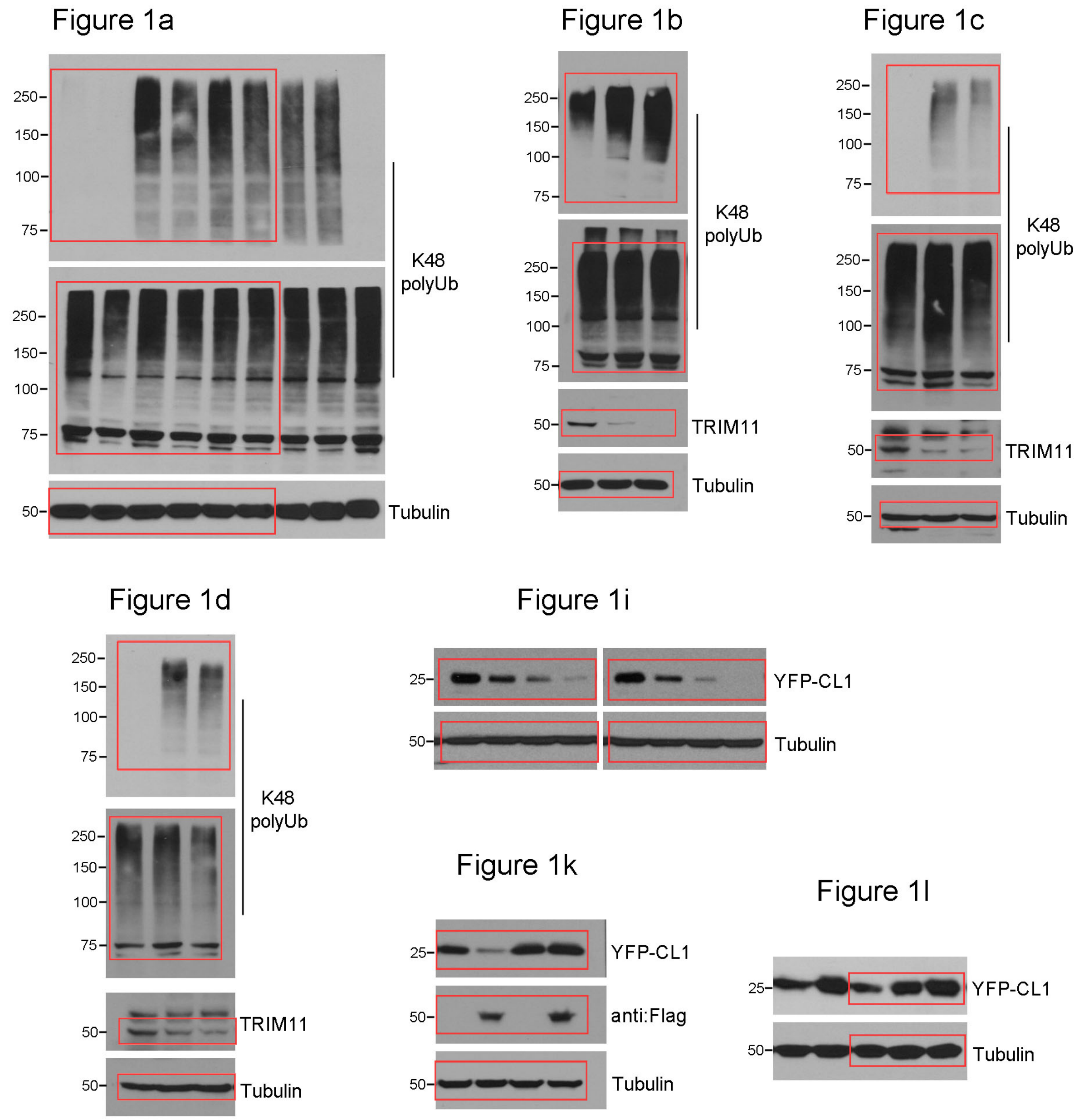

Supplementary Figure 10 | Uncropped western blot images related to Figures 2 and 3

Figure 2a

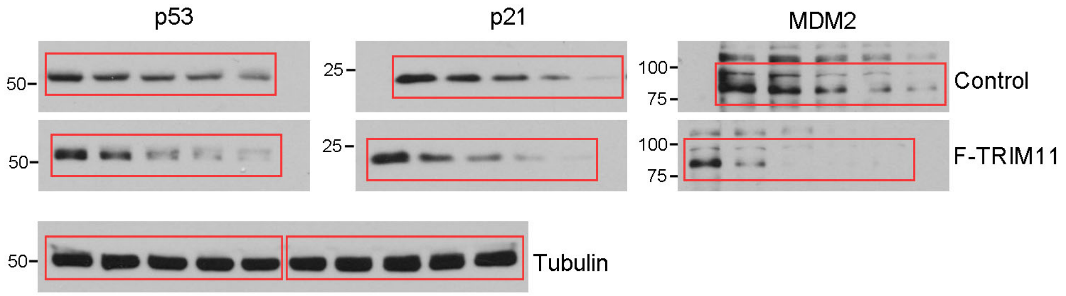

Figure 2c

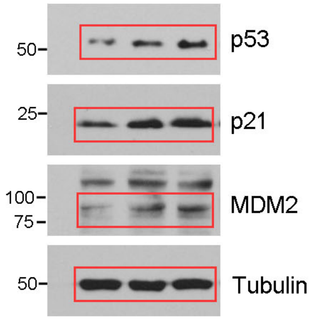

Figure 3b

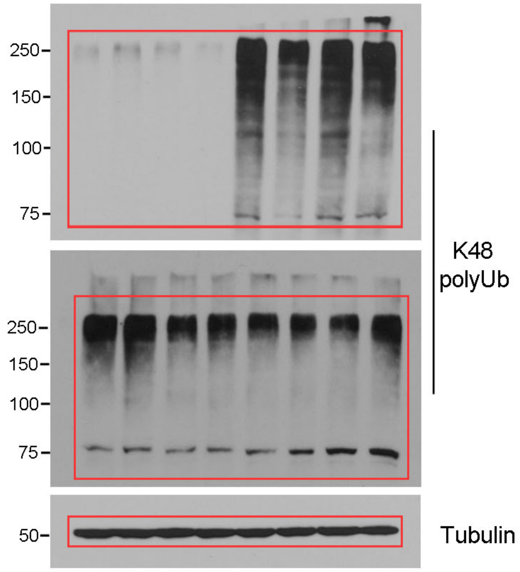

Supplementary Figure 11 | Uncropped western blots image related to Figure 4

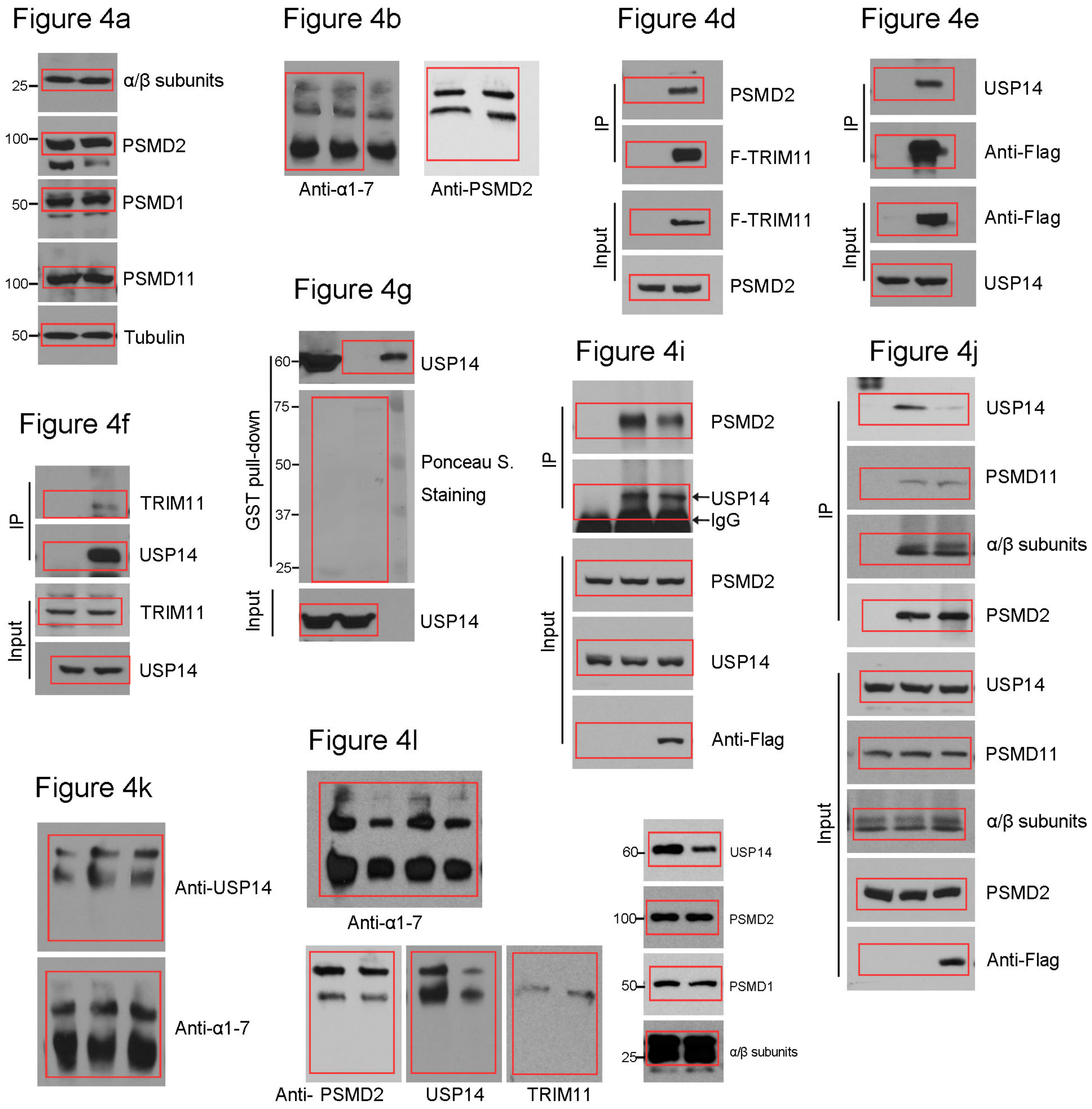

Supplementary Figure 12 | Uncropped western blots images related to Figure 5

Figure 5d

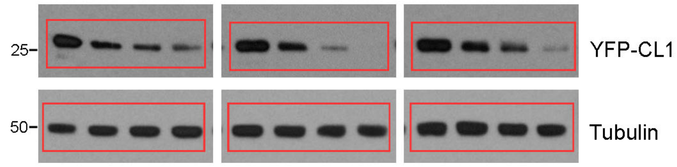

Figure 5g

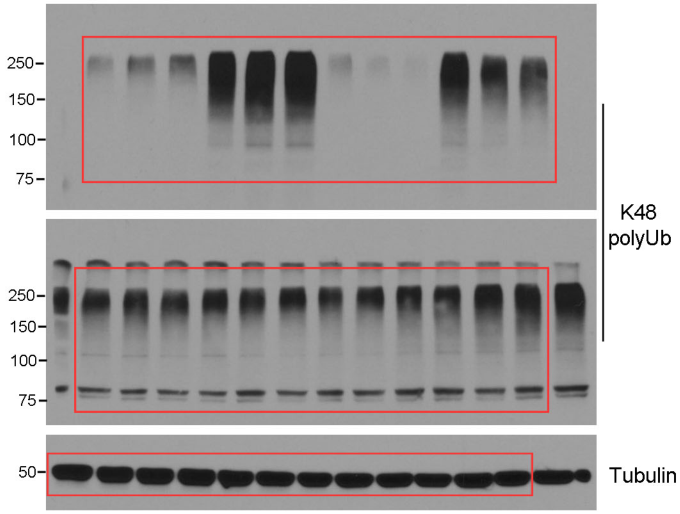

Figure 5h

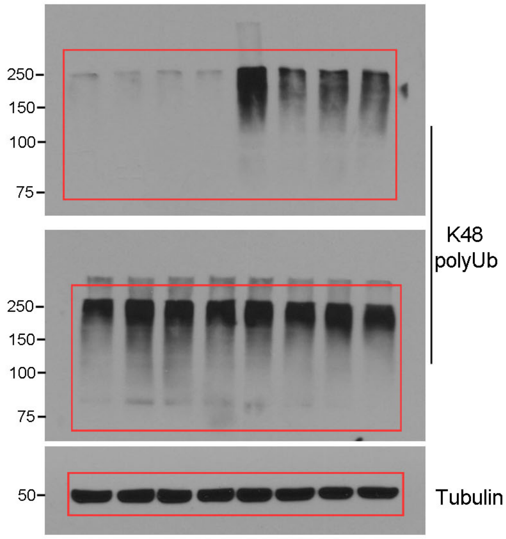

Supplementary Figure 13 | Uncropped western blots images related to Figure 6

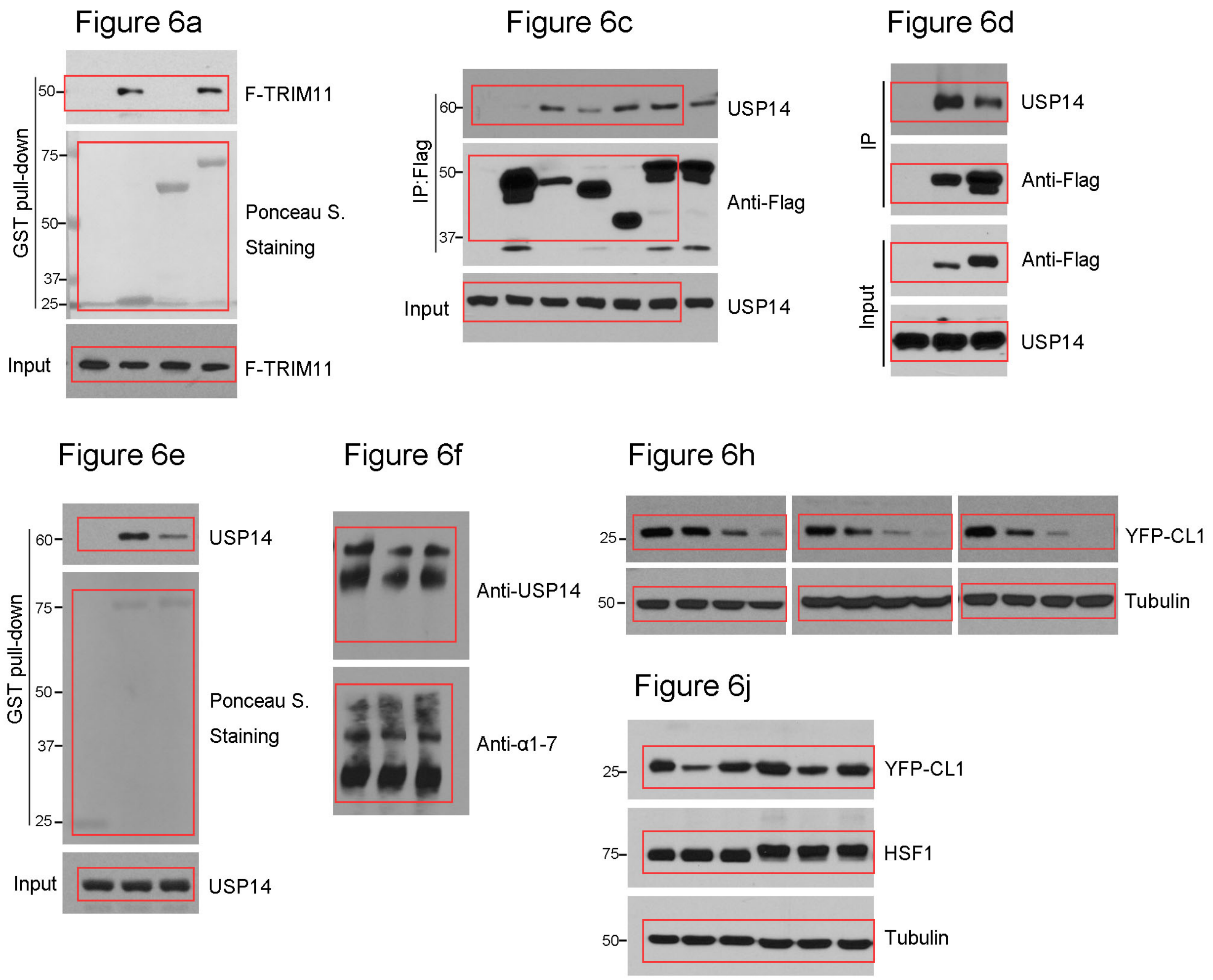

Supplementary Figure 14 | Uncropped western blots images related to Figure 7

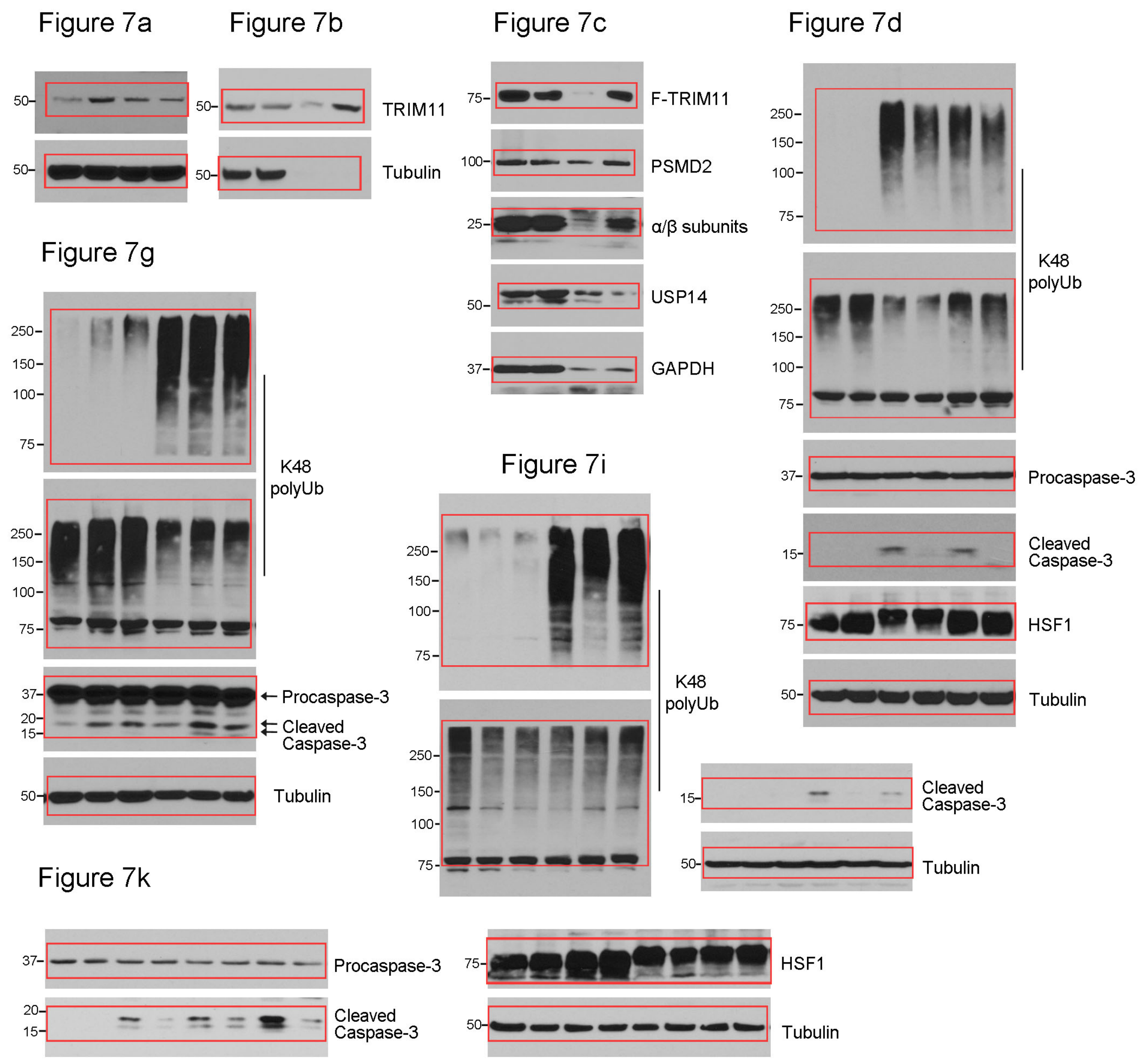

Supplement: Supplementary file 1 — Supplementary Information(PDF 4139 kb) [file 41467_2018_3499_MOESM1_ESM.pdf]
